# Functionally and structurally distinct fusiform face area(s) in over 1000 participants

**DOI:** 10.1016/j.neuroimage.2022.119765

**Published:** 2022-11-23

**Authors:** Xiayu Chen, Xingyu Liu, Benjamin J. Parker, Zonglei Zhen, Kevin S. Weiner

**Affiliations:** a Faculty of Psychology, Beijing Normal University, Beijing 100875, China; b State Key Laboratory of Cognitive Neuroscience and Learning, Beijing Normal University, Beijing 100875, China; c Helen Wills Neuroscience Institute, University of California, Berkeley, CA 94720, United States; d Department of Psychology, University of California, Berkeley, CA 94720, United States

**Keywords:** Fusiform face area, Multimodal MRI, Face selectivity, Cortical thickness, Myelination, Functional connectivity

## Abstract

The fusiform face area (FFA) is a widely studied region causally involved in face perception. Even though cognitive neuroscientists have been studying the FFA for over two decades, answers to foundational questions regarding the function, architecture, and connectivity of the FFA from a large (N>1000) group of participants are still lacking. To fill this gap in knowledge, we quantified these multimodal features of fusiform face-selective regions in 1053 participants in the Human Connectome Project. After manually defining over 4,000 fusiform face-selective regions, we report five main findings. First, 68.76% of hemispheres have two cortically separate regions (pFus-faces/FFA-1 and mFus-faces/FFA-2). Second, in 26.69% of hemispheres, pFus-faces/FFA-1 and mFus-faces/FFA-2 are spatially contiguous, yet are distinct based on functional, architectural, and connectivity metrics. Third, pFus-faces/FFA-1 is more face-selective than mFus-faces/FFA-2, and the two regions have distinct functional connectivity fingerprints. Fourth, pFus-faces/FFA-1 is cortically thinner and more heavily myelinated than mFus-faces/FFA-2. Fifth, face-selective patterns and functional connectivity fingerprints of each region are more similar in monozygotic than dizygotic twins and more so than architectural gradients. As we share our areal definitions with the field, future studies can explore how structural and functional features of these regions will inform theories regarding how visual categories are represented in the brain.

## 1. Introduction

Determining how visual categories are represented in the brain continues to be a major goal and a highly debated topic in cognitive neuroscience with many different proposed theories (Apurva et al., 2004; Behrmann and Plaut, 2013; Grill-Spector and Weiner, 2014; Haxby et al., 2001, 2011; Huth et al., 2012, 2016; Kanwisher, 2000, 2010; Kriegeskorte et al., 2008; Mahon and Caramazza, 2009; Malach et al., 2002; Martin, 2007; McGugin et al., 2012; Pitcher and Ungerleider, 2021; Tarr and Gauthier, 2000). Theoretical debates aside – for example, the ever-popular arguments between modular vs. distributed processing (Haxby et al., 2000, 2001, 2011; Kanwisher et al., 1997; Kanwisher, 2000, 2010), as well as the role of expertise (Gauthier et al., 1999, 2000; McGugin et al., 2012; Tarr and Gauthier, 2000) in the importance, emergence, and function of clustered and distributed category representations in ventral temporal cortex (VTC) – there is great interest in cortical networks selective for faces across species (Arcaro et al., 2019; Bell et al., 2011; Grill-Spector et al., 2017; Nasr et al., 2011; Pinsk et al., 2009; Silson et al., 2016, 2018; Tsao et al., 2008; Tsao and Livingstone, 2008). In humans, the fusiform face area (FFA; Kanwisher et al., 1997; Kanwisher, 2010) is a widely studied functional region located in VTC that is causally involved in face perception (Jonas et al., 2018; Jonas and Rossion, 2021; Parvizi et al., 2012; Rangarajan et al., 2014; Schalk et al., 2017). Nevertheless, even though the extended field has been studying the FFA for over two decades and despite great interest in the FFA in development (Cohen et al., 2019; Deen et al., 2017; Golarai et al., 2007; Gomez et al., 2017; Grill-Spector et al., 2008; Scherf et al., 2007, 2012, 2014), ageing (Park et al., 2012), and among patient populations (Avidan and Behrmann, 2021; Duchaine and Yovel, 2015; Golarai et al., 2010; Jonas and Rossion, 2021; Maher et al., 2019; Rossion, 2008; Rossion et al., 2003, 2018; Schalk et al., 2017), we still lack answers to foundational questions regarding the function and structure of the FFA from a large (N>1000) group of participants with analyses at the level of individual participants.

These gaps in knowledge persist for two main reasons. First, most human brain imaging studies perform analyses at the group level in which data are collapsed across participants and analyzed in volume space (previously referred to as “traditional neuroimaging methods”; Coalson et al., 2018). However, group-level functional maps often do not match the functional organization in individual participants. In fact, a recent review paper used the fusiform face complex (FFC) within the Human Connectome Project (HCP) multimodal parcellation atlas (MMP) proposed by Glasser et al. (2016a) as an example to illustrate this mismatch (Glasser et al., 2016a; Van Essen and Glasser, 2018). “FFC” was used to refer to the fact that at the group level, the authors were unable to subparcellate the complex into more than one area likely due to spatial blurring that occurs with group analyses. Second, studies performing analyses within individual participants manually define the FFA in each hemisphere, which while an arduous process, is still the most accurate method for defining functional regions in individual participants – even for primary sensory areas given recent findings (Benson et al., 2022) – compared to automated approaches. Consequently, given this manual and labor-intensive process, many studies interested in face processing at the level of individual participants suffer from relatively small sample sizes (typically in the ballpark between 10 and 50 participants; Çukur et al., 2013; Davidenko et al., 2012; Downing et al., 2006; Elbich and Scherf, 2017; Engell and McCarthy, 2013; Finzi et al., 2021; Gomez et al., 2015, 2017, 2018; Grill-Spector et al., 2004; Julian et al., 2012; Kay et al., 2015; Kietzmann et al., 2012; McGugin et al., 2014, 2015, 2016; Natu et al., 2016, 2019; Nordt et al., 2021; Parvizi et al., 2012; Pitcher et al., 2011; Rosenke et al., 2020, 2021; Scherf et al., 2017; Stigliani et al., 2015, 2019; Weiner et al., 2010, 2014, 2016, 2017; Weiner and Grill-Spector, 2010; countless others) because manually defining functional regions is time consuming.

Here, guided by classic multimodal criteria (Van Essen, 2003), we fill these gaps in knowledge by quantifying functional, architectural, and connectivity features of fusiform face-selective regions in 1053 participants included in the Human Connectome Project. To do so, we implemented a four-fold approach. First, we manually identified fusiform face-selective regions in all 2,106 hemispheres to determine incidence rates regarding how often a participant will have 0, 1, or 2 fusiform face-selective regions in either a left or right hemisphere in a large group of participants for the first time. Second, we extracted architectural (cortical thickness, myelination) features of each region. Third, we quantified functional (face selectivity) and connectivity (resting-state functional connectivity) features of each region. Fourth, we examined the similarity in spatial patterns of each functional, architectural, and connectivity feature between pairs of monozygotic (MZ) and dizygotic (DZ) twins included in the HCP dataset. As we share our areal definitions with the field, future studies can perform novel multimodal analyses that leverage the rich multimodal HCP dataset to explore how structural and functional features of these regions relate to cognitive and behavioral metrics also acquired in each participant.

## 2. Materials and methods

### 2.1. Data overview

HCP-Young Adult (HCP-YA, S1200 data release, 2017) data were used to define two face-selective regions on the fusiform gyrus (pFus-faces/FFA-1, mFus-faces/FFA-2) and to compare their i) cortical thickness, ii) myelination, iii) face selectivity, and iv) resting-state functional connectivity (RSFC) profiles. Additionally, spatial patterns of these structural and functional features in each region were compared between pairs of monozygotic (MZ) or dizygotic (DZ) twins. The HCP-YA includes behavioral and multi-modal MRI data from 1206 healthy young adult participants (i.e., S1200). After excluding the subjects with incomplete MRI scans or invalid MSMAll registration, 1053 participants (575 females, ages 22 to 37) were retained. Behavioral performance was not used to screen participants. Each participant completed structural MRI (sMRI), resting-state functional MRI (rfMRI), and task functional MRI (tfMRI) scans (Van Essen et al., 2013). Among them, there were 196 twin pairs (121 MZ twins and 75 DZ twins, M/F: 154/238) and 45 participants (31 females, ages 22 to 35) participated in test-retest reliability sessions. All participants provided written informed consent. MRI protocols were approved by the Institutional Review Board (IRB) of Washington University.

### 2.2. MRI acquisition

The HCP-YA MRI data were acquired on the HCP’s custom 3T Siemens Skyra scanner using a 32-channel head coil. T1-weighted (T1w) images were acquired using the 3D MPRAGE sequence (TR = 2400 ms, TE = 2.14 ms, voxel size = 0.7 mm isotropic, iPAT = 2). T2-weighted (T2w) images were acquired using the 3D SPACE sequence (TR = 3200 ms, TE = 565 ms, voxel size = 0.7 mm isotropic, iPAT = 2). Functional data were acquired using gradient-echo EPI sequence (TR = 720 ms, TE = 33.1 ms, voxel size = 2 mm isotropic, MB = 8). Four runs of rfMRI data were acquired for each participant from the HCP-YA, each of which were approximately 15 minutes. Details of the HCP-YA MRI acquisition can be found elsewhere (Barch et al., 2013; Glasser et al., 2013; Smith et al., 2013; Uğurbil et al., 2013).

### 2.3. Functional localizer

Face-selective regions were localized using a working memory task in which four stimulus types (faces, places, tools, and body parts) were presented in separate blocks (Barch et al., 2013). The localizer consisted of two runs, and each run contained eight task blocks (10 trials of 2.5 s each, for 25 s) and 4 fixation blocks (15 s each). Within each run, half of the task blocks used a 2-back working memory task and the other half implemented a 0-back working memory task. A 2.5 s cue indicated the task type at the start of the block. For each trial, the stimulus was presented for 2 s, followed by a 500 ms inter-trial interval (ITI).

### 2.4. Emotion processing paradigm

In each of two runs, participants were presented with 3 face blocks and 3 shape blocks (21 s each) (Barch et al., 2013). Each block, preceded by a 3 s task cue (“shape” or “face”), had 6 trials (2 s each, with a 1 s ITI). When the stimulus was presented, participants decided which of two faces/shapes presented on the bottom of the screen matched the face/shape at the top of the screen. The faces had either angry or fearful expressions.

### 2.5. MRI preprocessing

The MRI data of HCP-YA were preprocessed with the HCP minimal preprocessing pipelines (Glasser et al., 2013). The T1w and T2w images were used to i) reconstruct individual cortical surfaces, ii) estimate the T1w/T2w ratio (which is a measure of tissue contrast enhancement that is a proxy for myelination), and iii) cortical thickness. The individual surfaces and related maps were further registered to the standard fsLR surface via the multimodal surface matching (MSM) algorithm (Glasser et al., 2016a; Robinson et al., 2014). All functional images from individual participants were motion corrected, temporally filtered (highpass filter, cutoff = 2000 s for rfMRI data and 200 s for tfMRI data), spatially denoised via the ICA+FIX approach (for rfMRI only), and registered to the standard CIFTI grayordinate fsLR space using the MSM algorithm. The preprocessed task fMRI data were entered into a general linear model (GLM) to estimate fMRI activity at each vertex/voxel in each run with FSL (FMRIB’s Software Library, www.fmrib.ox.ac.uk/fsl) (Barch et al., 2013). The boxcar convolved with a double gamma hemodynamic response function, and its temporal derivative was used to model the BOLD responses. Linear contrasts were computed to estimate effects of interest (e.g., faces vs. others; faces vs. shapes). Fixed-effects analyses were conducted to estimate the average effects across runs within each participant.

The data used in this study were in the 32k_fs_LR space based on MSMAll registration, and no spatial smoothing was implemented.

### 2.6. Manual definition of mFus-faces/FFA-2 and pFus-faces/FFA-1 in over 1,000 participants

**Fig. 1.**
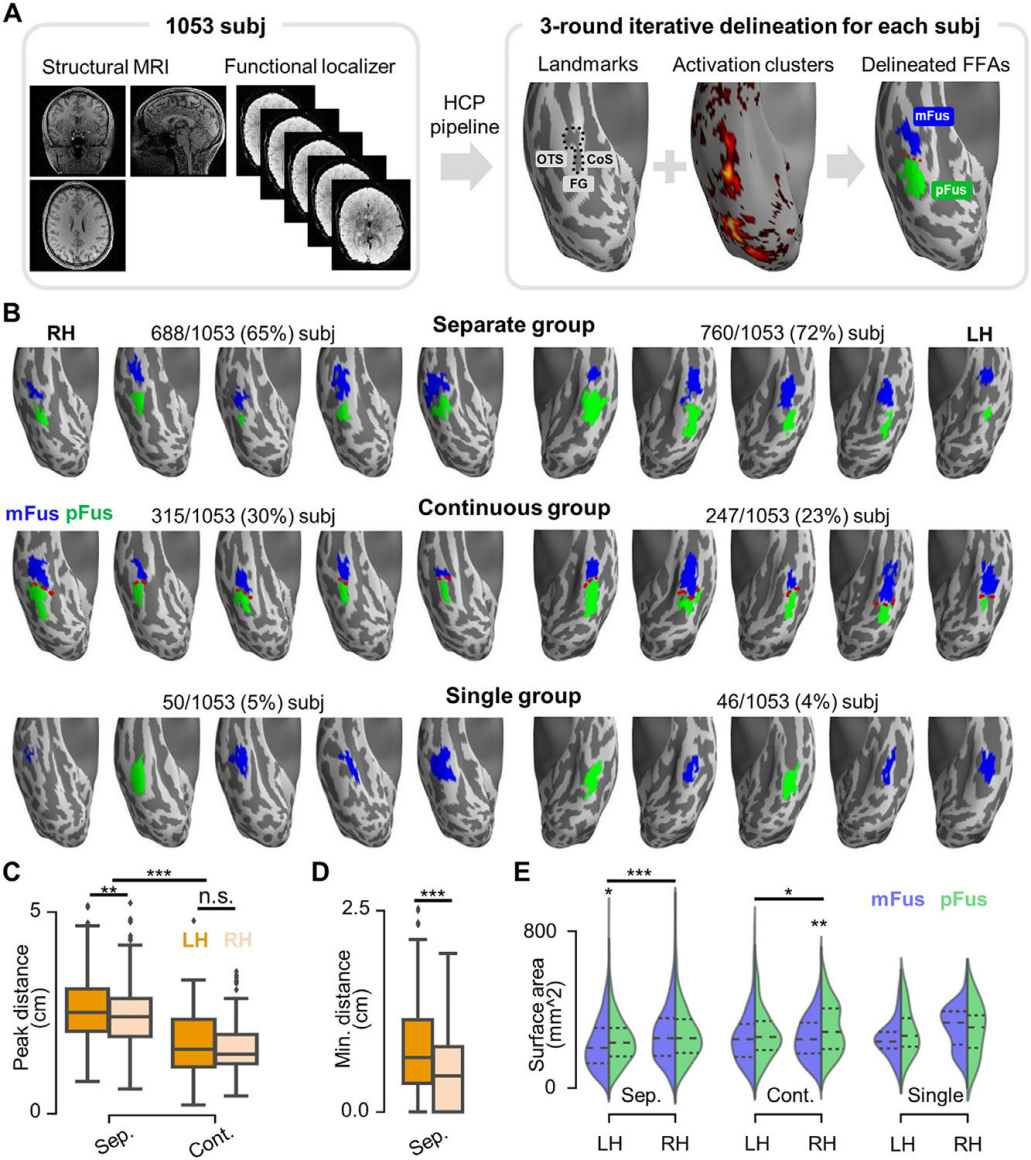
Three topological groups of face-selective regions on the lateral fusiform gyrus (FG) in over 1000 participants. (A) Face-selective regions were manually delineated on the lateral aspect of the FG in 1053 participants from the HCP using structural (left) and functional (right) data. By taking both individual cortical landmarks (OTS: occipito-temporal sulcus; CoS: collateral sulcus; MFS: mid-fusiform sulcus (black dotted line)) and face-selective activation clusters (faces versus others, Z>1.65, p<0.05, uncorrected) into account, face-selective regions were labeled as either mFus-faces/FFA-2 or pFus-faces/FFA-1 in each hemisphere based on previously published criteria differentiating the cortical location of these two regions. Specifically, pFus-faces/FFA-1 is located adjacent to the posterior extent of the MFS extending into the lateral FG and the nearby OTS, while mFus-faces/FFA-2 is located adjacent to the anterior tip of the MFS. A three round iterative delineation procedure was implemented for the definition of face-selective regions in each hemisphere (Materials and Methods). (B) Face-selective regions are depicted from 30 randomly chosen hemispheres (5 for each hemisphere and each group). Top row: separate group; Middle row: continuous group; Bottom row: single group. Incidence rates are included above each row for the RH and LH, respectively. (C) Cortical distance between the most face-selective vertices of the two face-selective regions in separate and continuous groups. (D) Cortical gap size between the two face-selective regions in the separate group, calculated as the minimum distance between them. (E) Surface areas of individual face-selective regions within the three groups. *p<0.05; **p< 0.01; ***p<0.001; n.s., not significant. LH: left hemisphere; RH: right hemisphere.

Face-selective regions on the lateral fusiform gyrus (FG) were manually delineated for each hemisphere and each participant based on individual, thresholded (Z>1.65, p<0.05, uncorrected) face-selective activation maps (faces versus others). From this thresholded map, regions of interest (ROIs) were labeled as either mFus-faces/FFA-2 or pFus-faces/FFA-1 based on previously published criteria differentiating the cortical location of the two regions relative to sulci within and surrounding the FG (Fig. 1A). Specifically, mFus-faces/FFA-2 is coupled with the anterior tip of the mid-fusiform sulcus (MFS) whereas pFus-faces/FFA-1 is located on the posterior aspect of the FG, extending into the occipito-temporal sulcus (Weiner, 2019; Weiner et al., 2014). To define each region, we implemented a three-pronged approach. First, author X.C. labeled each region manually on the individual thresholded face-selective map with customized software (FreeROI, https://github.com/BNUCNL/FreeROI). Second, author Z.Z. checked the regions and refined them together with X.C. Third, cognitive neuroanatomist K.S.W. finalized the regions.

Here, we used a liberal threshold (Z>1.65, p<0.05, uncorrected) for the main reason that we did not want to artificially inflate the “separate” group by using a strict threshold. Nevertheless, we recognize that thresholded statistical maps (i.e., t or z maps) are more susceptible to noise than those from gradient effect size (or beta) maps (Glasser et al., 2016b). However, previous research indicates that thresholded statistical maps produce reliable and reproducible face-selective regions within and across individuals despite differences in preprocessing choices (Engell and McCarthy, 2013; Julian et al, 2012; Kawabata Duncan and Devlin, 2011; Weiner et al., 2010, 2014; Weiner and Grill-Spector, 2010, 2011, 2012; Zhen et al., 2015). Thus, we defined face-selective regions based on thresholded z-statistic maps. As we share our definitions with the field, future studies can compare our definitions with those based on gradients of effect size maps.

### 2.7. Incidence rates and surface area of mFus-faces/FFA-2 and pFus-faces/FFA-1

Overall, we categorized the spatial organization of mFus-faces/FFA-2 and pFus-faces/FFA-1 into three types, or topological groups (Fig. 1B): separate, continuous, and single. The “separate” group consisted of two cortically distinct face-selective regions in a given hemisphere that were separated by a cortical gap. The “continuous” group consisted of two regions that were identifiable and contiguous, but could be separated based on previously proposed anatomical criteria based on cortical folding (Weiner, 2019; Weiner et al., 2014). The “single” group consisted of one region in which either mFus-faces/FFA-2 or pFus-faces/FFA-1, but not both, was identifiable in a given hemisphere. After determining these three groups, we summarized the incidence rate of each group by counting how many hemispheres were in each group. The surface area of each region was also quantified. A 3-way mixed ANOVA with hemisphere (left hemisphere [LH], right hemisphere [RH]; within-subject), group (continuous, separate; between-subject), and region (pFus-faces/FFA-1, mFus-faces/FFA-2; within-subject) as factors was conducted to test the differences of surface area of each region among the three groups.

### 2.8. Cortical distance between mFus-faces/FFA-2 and pFus-faces/FFA-1

Geodesic distance was used to quantify the cortical distance between pFus-faces/FFA-1 and mFus-faces/FFA-2 by using the tvb-gdist package (https://github.com/the-virtual-brain/tvb-gdist). Geodesic distance is the length of the shortest line between two vertices on a triangulated mesh in three dimensions, such that the line lies on the surface. The cortical distance between the most face-selective vertices (i.e., the activation peaks) of the two regions was calculated for hemispheres from continuous and separate groups and a 2-way mixed ANOVA was conducted to test the effects of hemisphere (LH, RH; within-subject) and group (continuous, separate; between-subject) on the distance. In addition, the cortical gap size between the two regions was measured for the separate group by calculating the minimum geodesic distance between the vertices of the two regions, and a paired t-test was performed to test the interhemispheric differences of the gap size.

### 2.9. The spatial consistency of mFus-faces/FFA-2 and pFus-faces/FFA-1 across groups

A group-specific probabilistic map was created for each fusiform face-selective region in each group (separate, continuous, single) to characterize the likelihood that a given vertex belongs to that region across the participants on whom the region had been identified. For each region, the spatial consistency was calculated as the spatial pattern similarity between each pair of group-specific probabilistic maps. Specifically, the spatial patterns in the overlapped portion of each pair of group probabilistic maps were extracted to compute the Pearson correlation coefficient.

### 2.10. Average cortical thickness and myelination of mFus-faces/FFA-2 and pFus-faces/FFA-1

We tested if pFus-faces/FFA-1 and mFus-faces/FFA-2 were anatomically distinct by calculating average cortical thickness and myelination values from each region in each individual. The mean thickness and myelination values were generated by averaging each measurement across all vertices within each region in each hemisphere and participant within each of the three groups. Two, 3-way mixed ANOVAs were conducted to further examine the effects of hemisphere (LH, RH; within-subject), group (contiguous, separate; between-subject), and region (pFus-faces/FFA-1, mFus-faces/FFA-2; within-subject) on the cortical thickness and myelination content, respectively.

### 2.11. Comparing face-selectivity between mFus-faces/FFA-2 and pFus-faces/FFA-1

As pFus-faces/FFA-1 and mFus-faces/FFA-2 are defined based on the HCP working memory task, we used face and shape conditions from the emotional processing task, which was also included in the HCP dataset, as an additional independent dataset to compare face selectivity between the two face-selective regions in each of the three groups. These data were acquired in nearly all participants (920/1053 participants) and were completely independent from the data used to define each face-selective region. Face selectivity was quantified as the average z-value of the contrast (faces vs. shapes) within each functional region in each individual participant. A 3-way mixed ANOVA with hemisphere (LH, RH; within-subject), group (continuous, separate; between-subject), and region (pFus-faces/FFA-1, mFus-faces/FFA-2; within-subject) as factors was conducted to test if pFus-faces/FFA-1 or mFus-faces/FFA-2 differed in their mean face-selectivity.

### 2.12. Comparing resting state functional connectivity profiles between mFus-faces/FFA-2 and pFus-faces/FFA-1

To quantify functional connectivity differences between these two face-selective regions, we considered three scales i) areal, ii) network, and iii) global. At the areal level, we quantified the resting-state functional connectivity (RSFC) from each FG face-selective region to each of the HCP MMP areas (Glasser et al., 2016a) except the FFC (which includes mFus-faces/FFA-2 and pFus-faces/FFA-1). In detail, for each participant, RSFCs between each face-selective region and each of the HCP MMP cortical areas were derived for each run by calculating Pearson correlation coefficients between their resting-state BOLD time courses, and then averaged across the four runs. At the network level, we characterized the connectivity of the two face-selective regions to the twelve large-scale resting-state networks (RSNs) by summarizing the RSFCs to all MMP areas into 12-dimension RSFC “fingerprints” according to the Cole-Anticevic Brain Network Parcellation (CAB-NP) (Ji et al., 2019). At the global level, we characterized the global brain connectivity (Cole et al., 2010) of each face-selective region by averaging RSFC values across the twelve large-scale networks. At both areal and network levels, paired t-tests were conducted to compare RSFCs of pFus-faces/FFA-1 and mFus-faces/FFA-2, and false discovery rate (FDR) corrections were conducted for the 358/12 tests in each hemisphere and each group (continuous or separate), respectively. At the global level, a 3-way mixed ANOVA with hemisphere (LH, RH; within-subject), group (continuous, separate; between-subject), and region (pFus-faces/FFA-1, mFus-faces/FFA-2; within-subject) as factors was conducted to test the inter-regional differences in connectivity.

### 2.13. Comparing spatial patterns of functional, architectural, and connectivity features of mFus-faces/FFA-2 and pFus-faces/FFA-1 between pairs of monozygotic and dizygotic twins

In addition to our previous analyses, we also aimed to compare spatial patterns of functional, architectural, and connectivity features of pFus-faces/FFA-1 and mFus-faces/FFA-2 between pairs of MZ and DZ twins. We were able to do so because a subset of the 1053 participants within the HCP dataset are from 121 MZ pairs and 75 DZ pairs. If MZ twins show more similar spatial patterns than DZ twins in an anatomical or functional feature, it indicates that genes contribute to that feature to some extent. To this end, we measured the spatial pattern similarity of each pair of twins with a Pearson correlation coefficient, which requires that the spatial masks (i.e., ROI matrices) are the same. For this, the maximum probability maps (MPMs; threshold = 0.25) of mFus-faces/FFA-2 and pFus-faces/FFA-1 were used as the group-level spatial masks for each participant in each twin pair. Specifically, the spatial patterns of face selectivity, thickness, and myelination of pFus-faces/FFA-1 and mFus-faces/FFA-2 were directly extracted from the MPM masks and the spatial pattern of RSFC of each face-selective region was characterized as the RSFC fingerprint between its MPM mask and the 12 RSNs. A 3-way mixed ANOVA with zygosity (MZ, DZ; between-subject), region (pFus-faces/FFA-1, mFus-faces/FFA-2; within-subject), and hemisphere (LH, RH; within-subject) as factors was conducted to statistically compare similarities in each functional (face selectivity), connectivity (RSFC), and architectural (thickness, myelination) feature.

### 2.14. Characterizing architectural, connectivity, and functional features of the cortical gap between mFus-faces/FFA-2 and pFus-faces/FFA-1

To further understand brain features of the cortical gap between pFus-faces/FFA-1 and mFus-faces/FFA-2, we implemented a fourfold approach. First, the cortical gap between pFus-faces/FFA-1 and mFus-faces/FFA-2 was automatically defined in each participant from the separate group by merging the vertices which fall in the FFC and between pFus-faces/FFA-1 and mFus-faces/FFA-2. Second, the average cortical thickness and myelination values across vertices were calculated respectively for pFus-faces/FFA-1, the cortical gap, and mFus-faces/FFA-2 in each participant. A 2 (hemisphere: LH, RH) × 3 (region: pFus-faces/FFA-1, gap, mFus-faces/FFA-2) repeated measures ANOVA was then conducted to examine if the cortical gap is different from the two face areas on each architectural feature. Third, the RSFCs from pFus-faces/FFA-1, the cortical gap, and mFus-faces/FFA-2 to the 12 RSNs were calculated in each participant and a 3-way repeated measures ANOVA with hemisphere (LH, RH), region (pFus-faces/FFA-1, gap, mFus-faces/FFA-2), and network (12 RSNs) as factors was conducted to examine if the cortical gap had a different RSFC fingerprint compared to pFus-faces/FFA-1 and mFus-faces/FFA-2. Fourth, the category-selective response to face, body, place, and tool conditions were examined using the test-retest working memory task fMRI data from HCP. Specifically, pFus-faces/FFA-1, the cortical gap, and mFus-faces/FFA-2 were defined on the original HCP test data and activation (beta) values of the four categories (face, body, place, and tool) were extracted from the independent, retest data. Only participants who had two separate face-selective regions were used in the analysis (N=27/28 for the left/right hemispheres). A 3-way repeated measures ANOVA with hemisphere (LH, RH), region (pFus-faces/FFA-1, gap, mFus-faces/FFA-2), and category (face, body, place, tool) as factors was conducted to examine the functional selectivity difference among the three regions.

## 3. Results

### 3.1. 95.44% of hemispheres have two face-selective regions on the FG

We manually delineated face-selective regions on the lateral aspect of the fusiform gyrus (FG) in 1053 participants (Fig. 1A) from the HCP and determined incidence rates regarding how often a hemisphere had 0, 1, or 2 FG face-selective regions in a large group of participants for the first time. At least one face-selective region, or “fusiform face area” (FFA), was identifiable in every hemisphere in each participant and 95.44% of hemispheres had two face-selective regions on the FG. The spatial organization of FG face-selective regions could be categorized into one of three different types, or topological groups, in a given hemisphere: separate, continuous, or single. A majority of hemispheres belonged to the separate group in which 68.76% of hemispheres (LH: 72.17%; RH: 65.34%) contained two face-selective regions that were separated by a cortical gap of several millimeters (Fig. 1B, top). In the continuous group, which consisted of 26.69% of cases (LH: 23.46%; RH: 29.91%), mFus-faces/FFA-2 and pFus-faces/FFA-1 were identifiable and contiguous, but could be separated based on previously proposed cortical folding criteria (Fig. 1B, middle). Specifically, mFus-faces/FFA-2 was identified as the functional region located adjacent to the anterior tip of the mid-fusiform sulcus (MFS), while pFus-faces/FFA-1 was identified as the functional region located adjacent to the posterior extent of the MFS extending into the lateral FG and the nearby occipito-temporal sulcus (Weiner, 2019; Weiner et al., 2014). In the single group, which consisted of less than 5% of cases (LH: 4.37%; RH: 4.75%), either mFus-faces/FFA-2 or pFus-faces/FFA-1, but not both, was identifiable in a given hemisphere based on the criteria just described (Fig. 1B, bottom). To test the effects of within-subject factors (i.e., hemisphere and region), we only included data from participants whose left and right hemispheres were both in the same continuous or separate group in the subsequent analyses.

In the continuous and separate groups, an average of 2.27 centimeters (based on the geodesic distance) separated the most face-selective vertices of pFus-faces/FFA-1 and mFus-faces/FFA-2 (Fig. 1C). A 2-way mixed ANOVA with hemisphere (LH, RH; within-subject) and group (continuous, separate; between-subject) as factors revealed that the distance increased when two cortically separate regions were present (*F*(1, 634)=182.53, *p*<.001, *η*^2^=.22, 90% confidence interval (CI) [.18, .27]). Furthermore, the distance between the most selective vertices was larger in the LH compared to the RH within the separate group (*F*(1, 634)=10.88, *p*=.001, *η*^2^=.02, 90% CI [.00, .04]), but not within the continuous group (*F*(1, 634)=1.06, *p*=.304, *η*^2^=.00, 90% CI [.00, .01]). Additionally, within the separate group, there was a 0.59-centimeter cortical gap (on average) between mFus-faces/FFA-2 and pFus-faces/FFA-1 (Fig. 1D; measured by the minimum distance between the vertices of the two regions). This cortical gap size was larger in the LH than that in the RH (*t*(529)=9.03, *p*<.001, *d*=.39, 95% CI [.30 .48]), which supports previous qualitative observations in a much smaller sample size (N=7; Weiner and Grill-Spector, 2010).

Surface area differences in FG face-selective regions were also revealed by a 3-way mixed ANOVA with hemisphere (LH, RH; within-subject), group (continuous, separate; between-subject), and region (pFus-faces/FFA-1, mFus-faces/FFA-2; within-subject) as factors (Fig. 1E). Specifically, pFus-faces/FFA-1 was slightly larger compared to mFus-faces/FFA-2 within the right continuous group (*F*(1, 634)=7.53, *p*=.006, *η*^2^=.01, 90% CI [.00, .03]) and left separate group (*F*(1, 634)=4.62, *p*=.032, *η*^2^=.01, 90% CI [.00, .02]). Moreover, in the separate group, both regions were larger in the RH compared to the LH (*Fs*(1, 634)>=24.23, *ps*<.001, *η*^2^>.03). In the continuous group, right pFus-faces/FFA-1 was larger than left pFus-faces/FFA-1 (*F*(1, 634)=6.51, *p*=.011, *η*^2^=.01, 90% CI [.00, .03]).

### 3.2. The spatial distribution of face-selective regions is stable across groups, while pFus-faces/FFA-1 is more face-selective than mFus-faces/FFA-2

**Fig. 2.**
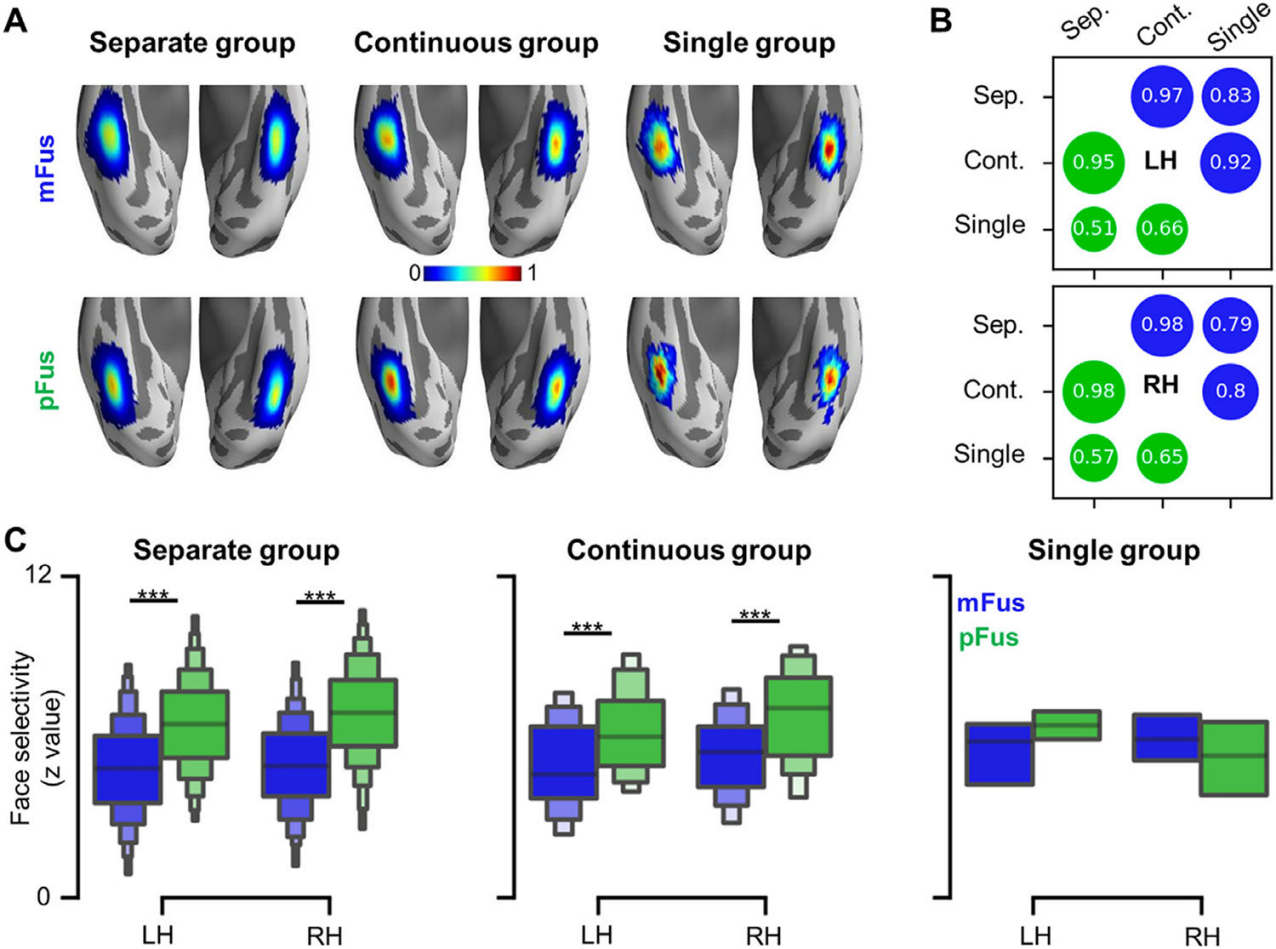
Spatial distribution and face selectivity of fusiform face-selective regions. (A) Probabilistic maps of face-selective regions in the three groups (separate, continuous, single). Top row: mFus-faces/FFA-2; Bottom row: pFus-faces/FFA-1. (B) Both face-selective regions showed high spatial consistency across groups in both hemispheres, measured by the Pearson correlation coefficient between the probabilistic maps of each pair of groups. The spatial consistency between the single group and either the continuous or separate group was lower because the probabilistic maps of the single group suffered from smaller sample sizes (Fig. 1 and Results for incidence rates). Blue circle: mFus-faces/FFA-2; Green circle: pFus-faces/FFA-1. (C) pFus-faces/FFA-1 (green) is more face-selective than mFus-faces/FFA-2 (blue) in both the separate and continuous groups. ***p<0.001. LH: left hemisphere; RH: right hemisphere.

A probabilistic map was created for each FG face-selective region in each group (Fig. 2A), which provided a vertex-wise description for the spatial distribution of each region. We found that both FG face-selective regions showed high spatial consistency across groups in both hemispheres (Fig. 2B). Specifically, the Pearson correlation coefficients between probabilistic maps from the separate and continuous groups are greater than 0.95. As expected, the spatial consistency between the single group and either the continuous or separate group was lower because the probabilistic maps of the single group suffered from smaller sample sizes.

After characterizing the stability of pFus-faces/FFA-1 and mFus-faces/FFA-2, we next tested if there were differences in face selectivity between the two regions. As pFus-faces/FFA-1 and mFus-faces/FFA-2 are defined based on the HCP working memory task, we used face and shape conditions from the emotional processing task, which was also included in the HCP dataset, as an independent dataset to compare face selectivity between the two face-selective regions in each of the three groups. Crucially, these data were acquired in nearly all participants and completely independent from the data used to define each face-selective region. We found that pFus-faces/FFA-1 is more face-selective than mFus-faces/FFA-2 (Fig. 2C). Specifically, a 3-way mixed ANOVA with hemisphere (LH, RH; within-subject), group (continuous, separate; between-subject), and region (pFus-faces/FFA-1, mFus-faces/FFA-2; within-subject) as factors revealed a region × group interaction (*F*(1, 548)=4.83, *p*=.028, *η*^2^=.01, 90% CI [.00, .03]). Further, we found that pFus-faces/FFA-1 is more face-selective than mFus-faces/FFA-2 in the continuous group (*F*(1, 548)=77.62, *p*<.001, *η*^2^=.12, 90% CI [.08, .17]) and separate group (*F*(1, 548)=659.38, *p*<.001, *η*^2^=.55, 90% CI [.50, .58]) in both hemispheres. Importantly, these effects were retained after regressing out temporal contrast-to-noise-ratio (CNR) (Fig. S2A).

### 3.3. mFus-faces/FFA-2 is cortically thicker and less myelinated than pFus-faces/FFA-1

Are there architectural differences between pFus-faces/FFA-1 and mFus-faces/FFA-2 that could serve as underlying anatomical substrates for the functional differences between these two regions? Two complementary approaches from previous studies suggest that pFus-faces/FFA-1 and mFus-faces/FFA-2 are likely architecturally distinct from one another. First, previous studies showed that microstructurally, pFus-faces/FFA-1 and mFus-faces/FFA-2 are located in different cytoarchitectonic territories (Gomez et al., 2018; Weiner et al., 2017). Second, additional work showed that cytoarchitectonic regions early in the visual processing hierarchy were cortically thinner and more myelinated than cytoarchitectonic regions positioned later in the visual processing hierarchy in which the expression of a sparse subset of genes contributed to these differences (Gomez et al., 2019). However, these studies combined data from living and post-mortem individuals to draw these conclusions. Thus, building on these previous findings, we tested if pFus-faces/FFA-1 and mFus-faces/FFA-2 were architecturally distinct by calculating average cortical thickness and myelination values from each region in each individual participant within a large group of participants for the first time.

**Fig. 3.**
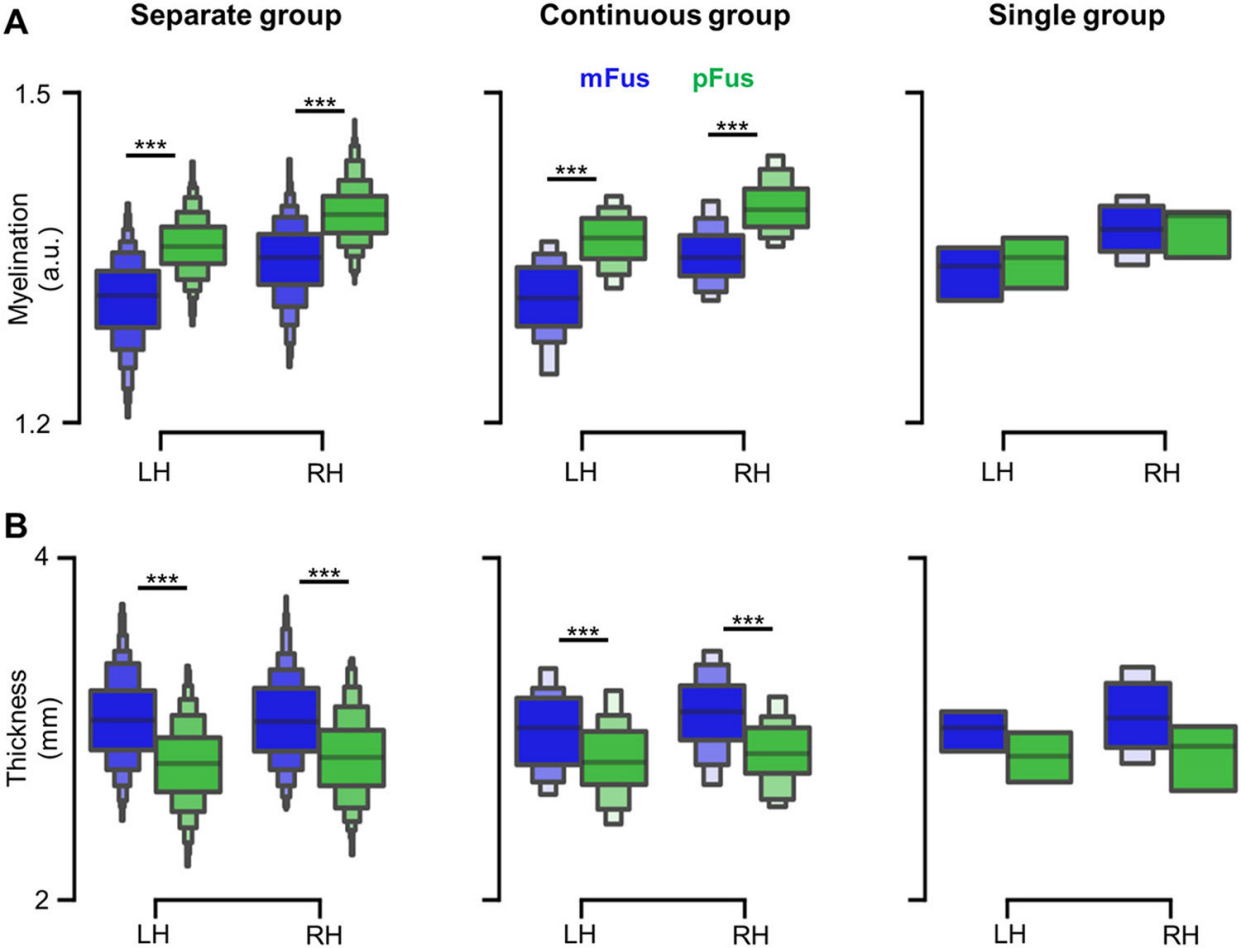
mFus-faces/FFA-2 is cortically thicker and less myelinated than pFus-faces/FFA-1. (A) pFus-faces/FFA-1 (green) has a higher myelin content than mFus-faces/FFA-2 (blue) in the separate and continuous groups. (B) mFus-faces/FFA-2 (blue) is cortically thicker than pFus-faces/FFA-1 (green) across groups. ***p<0.001. LH: left hemisphere; RH: right hemisphere.

This approach revealed that mFus-faces/FFA-2 is cortically thicker and less myelinated than pFus-faces/FFA-1 (Fig. 3). Specifically, two 3-way mixed ANOVAs with hemisphere (LH, RH; within-subject), group (contiguous, separate; between-subject), and region (pFus-faces/FFA-1, mFus-faces/FFA-2; within-subject) as factors revealed that i) pFus-faces/FFA-1 had more myelin content than mFus-faces/FFA-2 in the left (*F*(1, 634)=470.21, *p*<.001, *η*^2^=.43, 90% CI [.38, .47]) and right hemisphere (*F*(1, 634)=315.41, *p*<.001, *η*^2^=.33, 90% CI [.29, .38]) (Fig. 3A); ii) mFus-faces/FFA-2 was cortically thicker than pFus-faces/FFA-1 in both hemispheres and groups (all *Fs*(1,634)>28.79, all *ps*<.001, all *η*^2^>.04) (Fig. 3B). Additionally, although the difference between the two regions occurred at the spatial mean level, we could not find a sharp boundary of thickness or myelination that separated mFus-faces/FFA-2 or pFus-faces/FFA-1 at the gradient level in each individual (Fig. S3-4).

### 3.4. mFus-faces/FFA-2 and pFus-faces/FFA-1 have different functional connectivity “fingerprints”

**Fig. 4.**
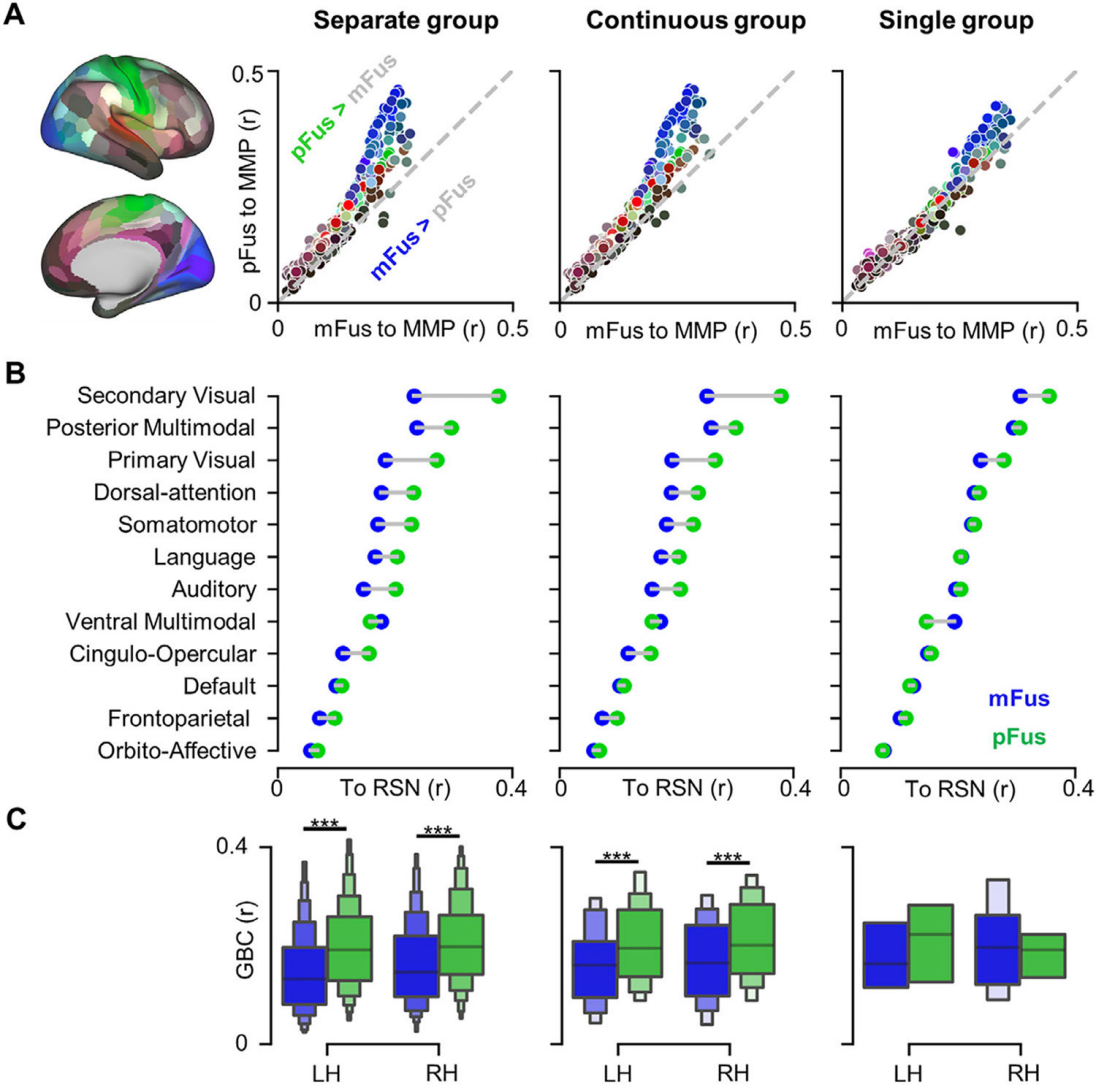
mFus-faces/FFA-2 and pFus-faces/FFA-1 have different resting-state functional connectivity (RSFC) “fingerprints”. (A) pFus-faces/FFA-1 showed stronger RSFC than mFus-faces/FFA-2 to most of 358 HCP MMP areas in the separate and continuous groups. After averaging the two hemispheres, 179 areas were displayed as points on each scatter plot with colors corresponding to the brain map at left. (B) pFus-faces/FFA-1 showed stronger RSFC than mFus-faces/FFA-2 to all of the 12 resting-state networks (RSNs) from (Ji et al., 2019) with the exception of the ventral multimodal network in the separate and continuous groups. RSFCs displayed here were merged across hemispheres. (C) Global brain connectivity (GBC) for each face-selective region, calculated as mean RSFCs of each face-selective region across RSNs. ***p<0.001. LH: left hemisphere; RH: right hemisphere.

To quantify potential functional connectivity differences between these two face-selective regions, we considered three scales: i) areal, ii) network, and iii) global. At the areal level, we quantified the intrinsic resting-state functional connectivity (RSFC) between face-selective regions and regions from the multimodal parcellation (MMP) of the human cerebral cortex by Glasser and colleagues (2016a). We found that pFus-faces/FFA-1 was more strongly connected to a majority of regions compared to mFus-faces/FFA-2 in both continuous and separate groups (all *t*s>1.99, all *p*s<.050, all *d*s>.07, FDR corrected; Fig. 4A). Furthermore, we found that mFus-faces/FFA-2 was more strongly connected to a relatively small number of regions compared to pFus-faces/FFA-1. In the continuous group, left mFus-faces/FFA-2 had stronger functional connectivity with 8 areas (all *t*s(227)>2.05, all *p*s<.047, all *d*s>.13, FDR corrected; Fig. S5A); right mFus-faces/FFA-2 had stronger functional connectivity with 18 areas (all *t*s(291)>2.11, all *p*s<.039, all *d*s>.12, FDR corrected; Fig. S5B). In the separate group, left mFus-faces/FFA-2 was more strongly connected to 17 areas (all *t*s(704)>2.38, all *p*s<.019, all *d*s>.08, FDR corrected; Fig. S6A), while right mFus-faces/FFA-2 was more strongly connected to 7 areas (all *t*s(636)>2.12, all *p*s<.037, all *d*s>.08, FDR corrected; Fig. S6B).

At the network level, the RSFCs of all MMP areas were summarized into 12-dimension RSFC “fingerprints” according to Cole-Anticevic Brain Network Parcellation (CAB-NP) (Ji et al., 2019). This approach revealed that these fingerprints were functionally distinct from one another when two regions were present (Fig. 4B). In both hemispheres and both groups, pFus-faces/FFA-1 showed stronger RSFC than mFus-faces/FFA-2 to all networks with the exception of the ventral multimodal network (all *t*s>2.42, all *p*s<.016, all *d*s>.14, FDR corrected); mFus-faces/FFA-2 showed stronger RSFC than pFus-faces/FFA-1 only in the ventral multimodal network (all *t*s>5.21, all *p*s<.001, all *d*s>.29, FDR corrected).

Finally, we examined global brain connectivity differences between pFus-faces/FFA-1 and mFus-faces/FFA-2 by averaging RSFC values across 12 networks separately for each region to summarize these effects across networks. A 3-way mixed ANOVA of the summarized RSFC with hemisphere (LH, RH; within-subject), group (separate, continuous; between-subject), and region (mFus-faces/FFA-2, pFus-faces/FFA-1; within-subject) as factors (Fig. 4C) revealed that at the global level, pFus-faces/FFA-1 had a higher RSFC than mFus-faces/FFA-2 (*F*(1, 587)=359.91, *p*<.001, *η*^2^=.38, 90% CI [.33, .43]). Importantly, this effect was retained after regressing out CNR (Fig. S2B).

### 3.5. Spatial patterns of face selectivity and functional connectivity, but not architectural features, in mFus-faces/FFA-2 and pFus-faces/FFA-1 were more similar between pairs of monozygotic than dizygotic twins

**Fig. 5.**
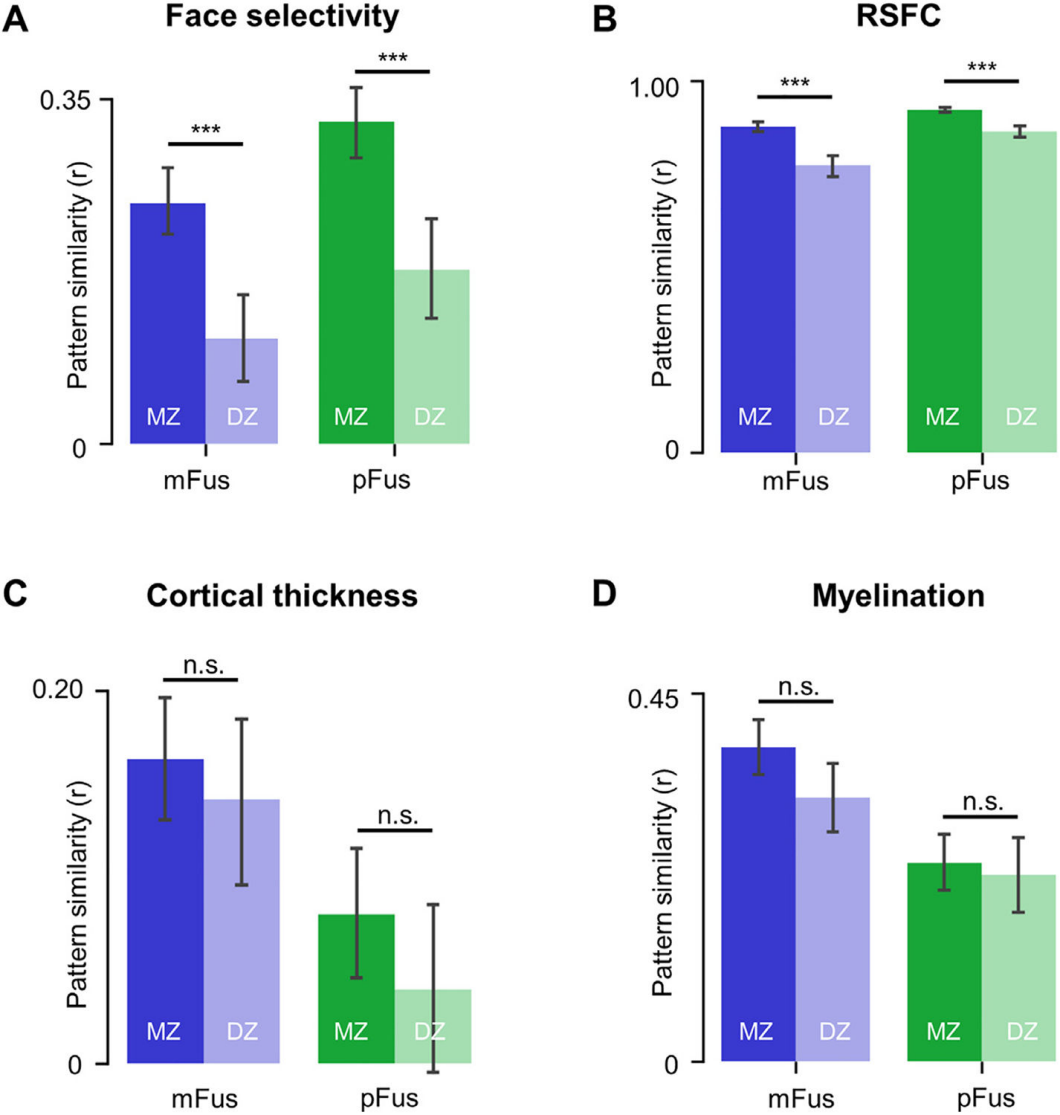
Spatial patterns of face selectivity and functional connectivity, but not architectural features, in pFus-faces/FFA-1 and mFus-faces/FFA-2 were more similar between pairs of monozygotic (MZ) than dizygotic (DZ) twins. (A) MZ twins showed significantly higher spatial pattern similarity in face selectivity than DZ twins for both face-selective regions. (B) MZ twins showed significantly higher spatial pattern similarity in resting-state functional connectivity (RSFC) than DZ twins for both face-selective regions. (C) MZ twins and DZ twins showed no significant differences in spatial pattern similarity of cortical thickness within both face-selective regions. (D) MZ twins and DZ twins showed no significant differences in spatial pattern similarity of myelination within both face-selective regions. Error bars indicate the 95% confidence interval; ***p<0.001; n.s., not significant.

Are there heritable components contributing to the functional, architectural, and connectivity differences between pFus-faces/FFA-1 and mFus-faces/FFA-2? Previous research indicates a genetic contribution to face processing ability (Wilmer et al., 2010; Wu et al., 2020; Zhu et al., 2010) and to the broad cortical morphology of category-selective regions in ventral temporal cortex (Abbasi et al., 2020). To test the above question that stems from these previous findings, we evaluated if spatial patterns of functional (face selectivity), connectivity (RSFC), and architectural (cortical thickness, myelination) features of pFus-faces/FFA-1 and mFus-faces/FFA-2 were more similar in monozygotic (MZ) than dizygotic (DZ) twins. We were able to do so because a subset of the 1053 participants within the HCP dataset are from 121 MZ pairs and 75 DZ pairs. The similarity of the spatial patterns from each twin pair was assessed by the Pearson correlation coefficient for each of the four functional or structural characteristics (Fig. 5). We found that the spatial patterns of face selectivity and functional connectivity, but not architectural features, of pFus-faces/FFA-1 and mFus-faces/FFA-2 were more similar between pairs of MZ than DZ twins. Specifically, significant main effects of zygosity were found for face selectivity (*F*(1, 194)=42.06, *p*<.001, *η*^2^=.18, 90% CI [.10, .26]) and for RSFC (*F*(1, 171)=44.74, *p*<.001, *η*^2^=.21, 90% CI [.12, .29]). Although there were interactions among zygosity, region, and hemisphere (all *F*s(1, 171)>4.01, all *p*s<=.047, all *η*^2^>.02) for RSFC, the effects of zygosity within each level of hemisphere and region were significant (all *F*s(1, 171)>18.53, all *p*s<.001, all *η*^2^>.09). Comparatively, there was no significant main effect of zygosity for either cortical thickness (*F*(1, 194)=2.27, *p*=.133, *η*^2^=.01, 90% CI [.00, .05]) or myelination (*F*(1, 194)=2.87, *p*=.092, *η*^2^=.02, 90% CI [.00, .05]).

### 3.6. The cortical gap is distinct from mFus-faces/FFA-2 and pFus-faces/FFA-1 based on architecture, connectivity, and selectivity

**Fig. 6.**
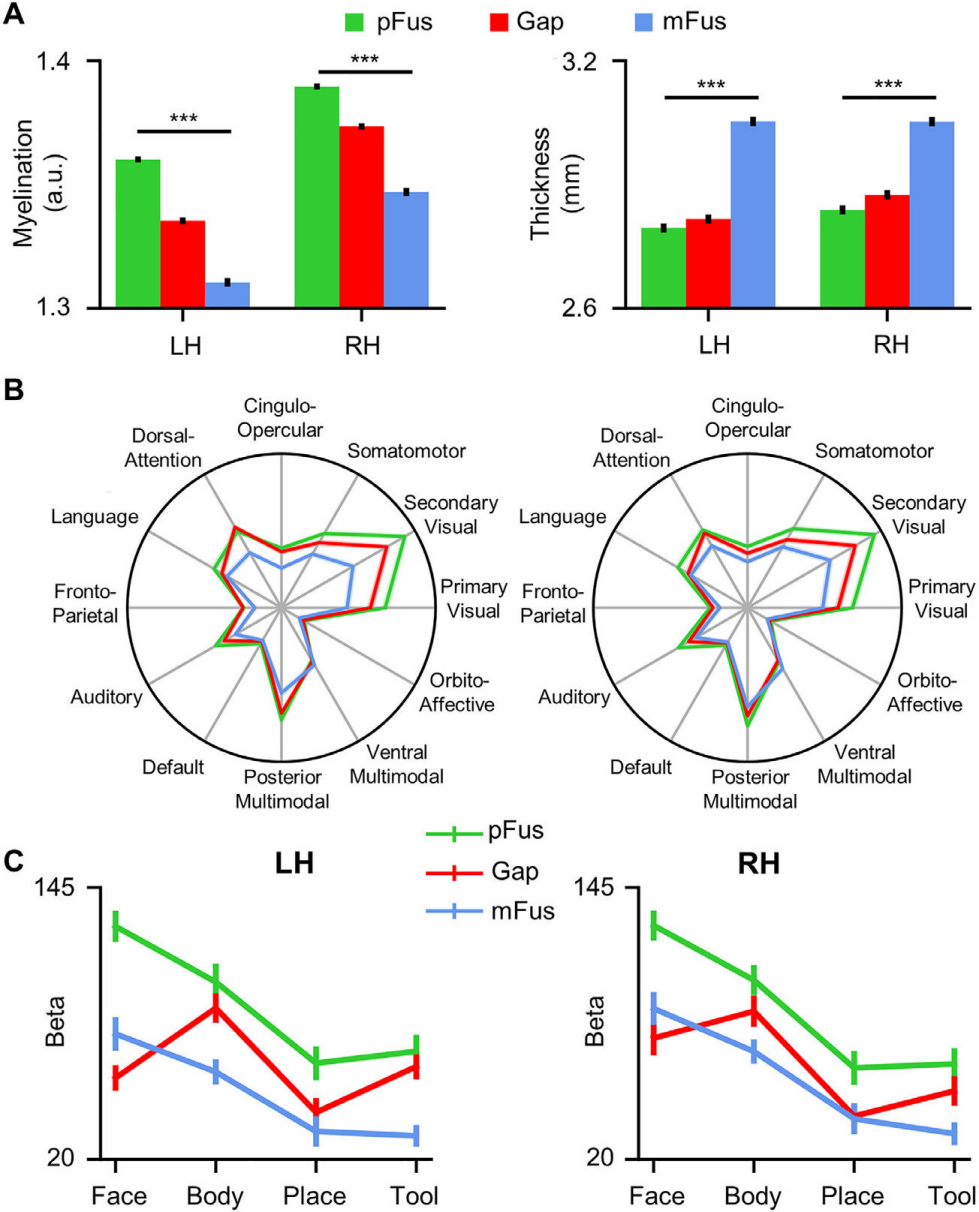
Architectural, connectivity, and functional features of the cortical gap between pFus-faces/FFA-1 and mFus-faces/FFA-2. (A) pFus-faces/FFA-1, the cortical gap, and mFus-faces/FFA-2 showed a gradient change in architectural features (i.e., myelination and thickness). (B) The cortical gap showed a different resting-state functional connectivity (RSFC) fingerprint compared to the two face-selective regions. (C) The cortical gap was functionally distinct from both pFus-faces/FFA-1 and mFus-faces/FFA-2 as it showed highest functional responses to bodies, not faces. LH: left hemisphere; RH: right hemisphere. Error bars represent +/− one SEM. ***p<0.001.

Finally, we examined the nature of the cortex that produces the gap between the two face-selective regions in term of its architectural, connectivity, and functional features. The cortical gap was automatically identified in each participant as the vertices which fall in the FFC and between pFus-faces/FFA-1 and mFus-faces/FFA-2. We reported three main findings. First, pFus-faces/FFA-1, the cortical gap, and mFus-faces/FFA-2 were architecturally different (Fig. 6A) and showed a gradient change in both myelination content (*F*(1.798, 951.034)=599.97, *p*<.001, *η*^2^=.53, 90% CI [.50, .56]) and cortical thickness (*F*(1.841, 973.884)= 363.17, *p*<.001, *η*^2^=.41, 90% CI [.37, .44]). Second, the cortical gap showed different RSFC to 12 RSNs compared to the two face-selective regions (Fig. 6B). A 3-way repeated measures ANOVA with hemisphere, region, and RSN as factors revealed a significant main effect of region (*F*(1.925, 1018.428)=377.49, *p*<.001, *η*^2^=.42, 90% CI [.38, .45]) and a significant interaction between region and RSN (*F*(8.085, 4276.828)=589.89, *p*<.001, *η*^2^=.53, 90% CI [.51, .54]). Third, the cortical gap showed a distinct response profile compared to both face-selective regions (Fig. 6C). This was consistent with previous results (Weiner and Grill-Spector, 2010, 2011), in which the cortical gap showed higher responses to images of bodies compared to images of faces and other categories. A 3-way repeated measures ANOVA with hemisphere, region, and category (face, body, place, tool) as factors revealed a significant main effect of region (*F*(2, 40)=30.37, *p*<.001, *η*^2^=.60, 90% CI [.41, .69]), a significant main effect of category (*F*(3, 60)=52.38, *p*<.001, *η*^2^=.72, 90% CI [.61, .78]) and a significant interaction between region and category (*F*(6, 120)=35.17, *p*<.001, *η*^2^=.64, 90% CI [.53, .68]). These findings indicate that the separation of pFus-faces/FFA-1 and mFus-faces/FFA-2 may be the result of another functional region, as occurs in frontal cortex for cases that area 55b forms a gap between the premotor eye field (PEF) and the frontal eye field (FEF) (Glasser et al., 2016a).

## 4. Discussion

Parcellating the cerebral cortex into areas continues to be a major goal in neuroscience. Over the last twenty-five years, the fusiform face area (FFA) is one of the most widely studied – and heavily debated – brain areas (Kanwisher, 2010, 2017; Kanwisher et al., 1997). In addition to many theories proposed to explain how and why humans and other mammals have neural responses selective for faces, researchers also debate if the FFA is one contiguous area or not. However, these previous studies have suffered from small sample sizes (often between 10 and 50 participants). Here, we defined 4116 face-selective regions on the fusiform gyrus (FG) in 1053 participants and showed that 95.44% of hemispheres have not one, but two, face-selective regions on the FG that are dissociable based on functional, architectural, and connectivity features. Additionally, we showed that the spatial patterns of face selectivity and functional connectivity are more highly correlated in monozygotic than dizygotic twins, which was surprisingly not the case for architectural features such as cortical thickness and myelination. Below, we consider these results in the context of i) future studies interested in the structure and function of face-selective regions on the FG, ii) individual differences in anatomy, face selectivity, and face perception, iii) understanding the complex relationship among genetics, anatomical gradients, and functional gradients, as well as how that relationship relates to perception, and iv) group averages vs. individual differences in neuroimaging studies.

### 4.1. Implications for future studies interested in the structure and function of face-selective regions on the FG

For more than a decade, dozens of studies have identified at least two face-selective regions on the FG (Çukur et al., 2013; Davidenko et al., 2012; Elbich and Scherf, 2017; Engell and McCarthy, 2013; Finzi et al., 2021; Gomez et al., 2015, 2017, 2018; Julian et al., 2012; Kay et al., 2015; Kietzmann et al., 2012; McGugin et al., 2014, 2015, 2016; Natu et al., 2016, 2019; Nordt et al., 2021; Parvizi et al., 2012; Pinsk et al., 2009; Rosenke et al., 2020, 2021; Scherf et al., 2017; Stigliani et al., 2015, 2019; Weiner et al., 2010, 2014, 2016, 2017; Weiner and Grill-Spector, 2010; Zhen et al., 2015) in addition to other face-selective regions in the core and extended systems of face processing (Haxby et al., 2000). Yet, to our knowledge, only two of these studies included more than 100 participants (N=121, Engell and McCarthy, 2013; N=202, Zhen et al., 2015) with the goal of generating probabilistic atlases. Critically, these two studies did not report individual differences in the structure or function of separate FG face-selective regions and the sample size was still a small percentage of that used in the present study. Here, we extend these previous studies by defining FG face-selective regions in over 1000 participants and show that the more posterior pFus-faces/FFA-1 is cortically thinner and more myelinated than the more anterior mFus-faces/FFA-2. Additionally, pFus-faces/FFA-1 is more face-selective with stronger functional connectivity to other cortical networks than mFus-faces/FFA-2.

Together, these results are surprising considering that it is widely accepted that identifying a single FFA is the norm, not the exception. Yet, our results empirically support the opposite in the largest group of manually defined face-selective regions on the FG to date (to our knowledge). For the 26.69% of hemispheres in the continuous group, the most likely factor contributing to the continuity of the regions is the spatial coarseness of the BOLD signal. That is, there is likely a cortical gap in these individuals, but the coarseness of the spatial spread of the BOLD signal causes the two regions to blur together. Indeed, the measured anatomical and functional differences between pFus-faces and mFus-faces was comparable in the continuous and separate groups. Additionally, since there is a relationship between the mid-fusiform sulcus (MFS) and the location of face-selective regions as identified in our previous work (Weiner et al., 2014), it’s likely that the morphology of the MFS (e.g., depth, length, etc) correlates with the size of the gap between face-selective regions. For example, the MFS can be as short as under 3 mm or as long as 7 cm (Weiner et al., 2014; Miller et al., 2020). Thus, the cortical gap between pFus-faces/FFA-1 and mFus-faces/FFA-2 may increase in the cases of a long MFS and decrease in the cases of a short MFS. Testing this hypothesis is now possible in a large group of participants, but will require the manual definition of the MFS in each hemisphere and participant, which can be examined in future studies. Additionally, our ongoing work (Parker et al., 2022) shows that there are differences in MFS length between individuals with Developmental Prosopagnosia and Neurotypical Controls and that MFS length is related to face processing ability, particularly in the right hemisphere. Similarly, for the less than 5% of hemispheres in the single group, we are able to examine if the presence or absence of either mFus-faces/FFA-2 or pFus-faces/FFA-1 is due to atypical sulcal patterns by defining the MFS in each individual to see if the sulcal variability of the MFS is greater in those with either mFus-faces/FFA-2 or pFus-faces/FFA-1 compared to those individuals who have both face-selective regions, which is an important topic for future research.

The present findings in combination with previous findings showing cytoarchitectonic (Weiner et al., 2017) and functional differences between these two regions (Kay et al., 2015; Weiner et al., 2010; Weiner and Grill-Spector, 2013), indicate that our findings are not just a matter of splitting one FFA into two. Instead, a majority of hemispheres contain two face-selective regions on the FG that are dissociable based on functional, architectural, and connectivity features. Thus, a goal of future empirical studies is to test for further functional differences between these regions, as well as similarities and differences in their anatomical connectivity. Future theoretical and computational work should also consider the FFA as two distinct regions in their models, as well as a third region in the anterior FG that is often immeasurable with fMRI due to methodological limitations (Jonas and Rossion, 2021). Additionally, even though FG face-selective regions are most often non-contiguous, the two regions together may constitute a functionally distinct system separate from other face-selective regions as suggested previously (Kanwisher, 2010) or perform the same function under certain task conditions despite the structural and functional differences identified here (the idea of “degeneracy”; Price and Friston, 2002; Edelman and Gally, 2001), both of which can be tested in future studies.

### 4.2. Genetics, anatomical gradients, and functional clusters on the human FG: Perceptual consequences?

Recent research indicates systematic relationships among gradients of genetic expression (e.g. transcriptomics) relative to architectural (e.g. cortical thickness and myelination) cortical features (Burt et al., 2018; Gomez et al., 2019). Additionally, recent findings also show that genetic expression in the brain is consistent with broad spatial trends that align well with network and connectomic architecture (Fornito et al., 2020), as well as functional maps within cortical areas (Gomez et al., 2021). The present results add additional novel insights to these previous findings. For example, even though there is a relationship among transcriptomics, cortical thickness, and myelination in the FG and more broadly across the visual processing hierarchy in humans (Gomez et al., 2019), there is a stronger correlation in MZ than DZ twins for face selectivity and functional connectivity properties of FG face-selective regions, but not cortical thickness and myelination. The latter finding indicates the utility of using different types of complementary data to improve our understanding of the complex relationship among genetics, anatomical gradients, and functional representations (gradients, maps, and clusters) in the human brain. As previous research shows genetic contributions also to face perception (Wilmer et al., 2010; Zhu et al., 2010) and the neural processing of faces (Abbasi et al., 2020; Brown et al., 2012), future studies can examine genetic contributions relating the structural and functional features of these FG face-selective regions to face processing ability.

For instance, does genetic expression contribute to the number of face-selective regions on the FG, which in turn, contributes to face processing ability? More broadly, what are the behavioral implications for only having one of these face-selective regions on the FG – or none at all? For example, there is recent causal evidence showing that electrical brain stimulation (EBS) to mFus-faces/FFA-2 results in deficits in naming faces, while EBS to pFus-faces/FFA-1 results in face-specific perceptual distortions (Schrouff et al., 2020). Such a result suggests that only having either mFus-faces/FFA-2 or pFus-faces/FFA-1 could have an effect on neural representations of either faces themselves in pFus-faces/FFA-1 or the integration of information about person identity in mFus-faces/FFA-2, which can be further examined in future studies. Additional recent findings also suggest that anatomical and morphological features of each region is related to face perception. For example, McGugin and colleagues (2016) showed that cortical thickness of pFus-faces/FFA-1 contributed more to behavioral performance on a face processing task than did mFus-faces/FFA-2 (McGugin et al., 2016). Additionally, the size of pFus-faces/FFA-1 was more tightly linked to behavior on a face processing task than the size of mFus-faces/FFA-2 (Elbich and Scherf, 2017). The combination of these causal and correlational results are consistent with the present results showing that pFus-faces/FFA-1 is more face-selective than mFus-faces/FFA-2. Taken together, the present findings lay the foundation for future work and mechanistic models linking genetics to face processing relative to underlying functional and structural differences between mFus-faces/FFA-2 and pFus-faces/FFA-1.

### 4.3. Averages vs. individual differences in neuroimaging studies

**Fig. 7.**
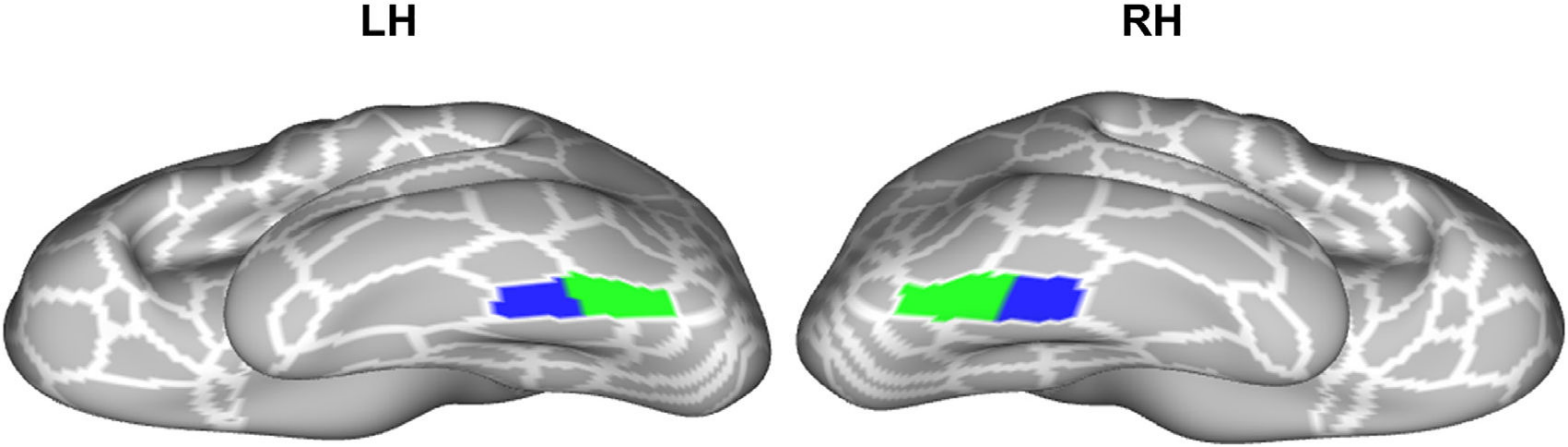
Empirical parcellation of FFC into pFus-faces/FFA-1 and mFus-faces/FFA-2 at the level of individual participants. Inflated cortical surface reconstructions of the left and right hemispheres are in 32k_fs_LR space. White lines are outlines of areas in the HCP MMP Atlas. Blue and green shaded areas indicate the new parcellation of area FFC into mFus-faces/FFA-2 (blue) and pFus-faces/FFA-1 (green), which was conducted at the level of individual participants and then summarized as a maximum probability map (threshold = 0).

A continued debate in the broader neuroimaging field is the balance between averages and group analyses compared to individual differences and analyses at the level of individual participants (Coalson et al., 2018; Friston et al., 2006; Genon et al., 2022; Gratton et al., 2022; Marek et al., 2022; Poldrack et al., 2015; Rosenberg and Finn, 2022; Saxe et al., 2006; Van Essen and Glasser, 2018). Directly related to this debate and the present findings, Van Essen and Glasser (2018) qualitatively showed that a group definition of the FFA (or what they referred to as a “strip-like” fusiform face complex, FFC) defined using the same dataset as used here does not align well with individual differences in the definition of face-selective regions on the FG in individual hemispheres. This observation is consistent with the present results showing that a majority of participants have two cortically distinct face-selective regions on the mid and posterior FG and even when there is one “strip-like” activation, it can be subdivided into two components that are functionally and architecturally distinct from one another with different functional connectivity profiles. Based on these results, we provide an empirical modification of the proposed FFC definition within the HCP MMP atlas – importantly, this modification is at the level of individual participants, which we share with the field (Fig. 7). This empirical modification is consistent with recent results that also propose modifications to other areal definitions in the HCP MMP atlas (Assem et al., 2020).

Moving forward, then, how do we i) strike a balance between group averages and individual differences (when both are necessary and complement one another) and ii) overcome the fact that defining regions of interest (ROIs) manually is monotonous, requires expertise, typically limits sample sizes, and limits the cortical expanse a particular study can explore? Here, we propose that a deep learning approach implemented previously on just the cortical anatomy, could also be implemented on functional definitions to improve the accuracy of automated definitions of functional brain regions in individual participants. Specifically, two recent studies (Borne et al., 2020; Lyu et al., 2021) used deep learning approaches to define sulci in individual participants with significant success. Each study first used many trained raters to manually define thousands of sulci and then trained and tested deep learning algorithms to label each sulcus. The algorithms accurately defined all sulci, but were the most accurate for deeper sulci that often had larger surface areas. This would suggest that once functional regions are manually defined in individual participants, the same algorithms could be trained, tested, and used to define functional regions in new participants. As the algorithms often improve as more data are used for training, functional ROIs defined in large, freely available datasets such as the multimodal data of the HCP at 3T and the retinotopy data of the HCP at 7T are good starting points for future studies to test the feasibility of this proposal. If successful, this approach would allow relatively automated approaches for accurate definitions of functional regions in individual participants – we use “relatively” here because the algorithms will first need to be trained on manually defined functional regions. In the interim, as we share our definitions with the field, future studies can perform novel multimodal analyses that leverage the rich multimodal HCP dataset to explore how anatomical and functional features of these face-selective regions relate to cognitive and behavioral metrics also acquired in each participant without needing the expertise to define each region manually. Finally, this approach also does not solve the balance between group analyses and analyses in individual participants for tasks, behaviors, and cognitive phenomena for which cortical regions and networks remain unknown.

## 5. Conclusion

In sum, we examined individual differences of fusiform face area(s) in a large group (N>1000) of participants for the first time. Our results show that identifying a single FFA is actually the exception, not the norm as described in the broader literature. Instead, it is most common to identify two face-selective regions on the lateral FG that are 2.27 cm apart on average between the most face-selective vertices, as well as are dissociable based on functional, architectural, and connectivity features. This organization of clustered regions or patches as opposed to a single larger area aligns well with face-selective patches identified in other species, such as macaques. Additionally, spatial patterns of functional (face selectivity) and connectivity (RSFC) features are more highly correlated in monozygotic compared to dizygotic twins, while architectural features (cortical thickness, myelination) are not. Future studies can leverage the fact that we are sharing our 4,116 manual areal definitions with the field to further explore how functional and structural features of these regions relate to cognitive and behavioral metrics also acquired in each participant within the rich multimodal HCP dataset.

## Supplementary Material

1

## Data Availability

The customized FreeROI toolbox, which is specifically developed to define subject-specific functional ROI (region of interest), can be found at: https://github.com/BNUCNL/FreeROI. The code for evaluating the functional, architectural, and connectivity features of the ROIs is available at: https://github.com/BNUCNL/HCP_FFA.

## Data availability

Multi-modal MRI data are provided by the Human Connectome Project, WU-Minn Consortium (Principal Investigators: David Van Essen and Kamil Ugurbil; 1U54MH091657) funded by the 16 NIH Institutes and Centers that support the NIH Blueprint for Neuroscience Research and by the McDonnell Center for Systems Neuroscience at Washington University. The data can be downloaded from Connectome DB (https://db.humanconnectome.org). The individual fusiform face-selective regions and associated probabilistic maps defined in this study are available both at http://www.brainactivityatlas.org and at BALSA (https://balsa.wustl.edu/study/x87Zk).
