# Supplementary material for: Functionally and structurally distinct fusiform face area(s) in over 1000 participants
Source: Neuroimage. Author manuscript; Available in PMC 2023 Jan 31. (PMC9889174; doi:10.1016/j.neuroimage.2022.119765)
Supplement: 1 [file NIHMS1867082-supplement-1.docx]

**SUPPLEMENTARY INFORMATION**

**The influence of fMRI dropout 2**

**Average and individual maps of myelin and thickness 4**

**Visualization of RSFC differences at the area level 6**

**Supplementary References 8**

*The influence of fMRI dropout*

Given that fusiform face-selective regions are located within portions of the inferior temporal lobe that are located near MR artifacts related to air and draining veins (Weiner and Grill-Spector, 2012, 2013), we used the temporal contrast-to-noise-ratio (CNR) of pFus-faces/FFA-1 and mFus-faces/FFA-2 to examine the influence of fMRI artifacts on our findings with a two-fold approach. First, the CNR was calculated for each vertex by dividing BOLD variance (BOLDVar) by unstructured noise variance (UnstructNoiseVar) for each rfMRI run. Specifically, UnstructNoiseVar was calculated as the standard deviation over the timeseries after regressing out the signal spatial ICA component timeseries from the cleaned resting state timeseries; BOLDVar was calculated as the difference between the standard deviation of the cleaned resting state timeseries and the UnstructNoiseVar. Second, the CNR of a region was calculated by averaging CNRs across vertices in that region, and then, across all runs for each participant. By comparing the CNRs across hemispheres and participants, we found a significant difference between pFus-faces/FFA-1 and mFus-faces/FFA-2 (*t*(4114)=26.62, *p*<.001, *d*=.83, 95% CI [.77, .89]) (Fig. S1). Nevertheless, when including CNR as a regressor, the functional differences between the two regions remained (Fig. S2). That is, two 3-way mixed ANOVAs with hemisphere (LH, RH; within-subject), group (continuous, separate; between-subject), and region (pFus-faces/FFA-1, mFus-faces/FFA-2; within-subject) as factors revealed that i) pFus-faces/FFA-1 is more face-selective than mFus-faces/FFA-2 (*F*(1, 548)=368.19, *p*<.001, $\eta^{2}$=.40, 90% CI [.35, .45]) and ii) pFus-faces/FFA-1 had a higher global brain connectivity than mFus-faces/FFA-2 both in the continuous group (*F*(1, 587)=170.06, *p*<.001, $\eta^{2}$=.22, 90% CI [.18, .27]) and separate group (*F*(1, 587)=577.48, *p*<.001, $\eta^{2}$=.50, 90% CI [.45, .54]). These results indicated that the CNR differences cannot fully account for the substantial differences between the two fusiform face-selective regions in both face selectivity and functional connectivity.


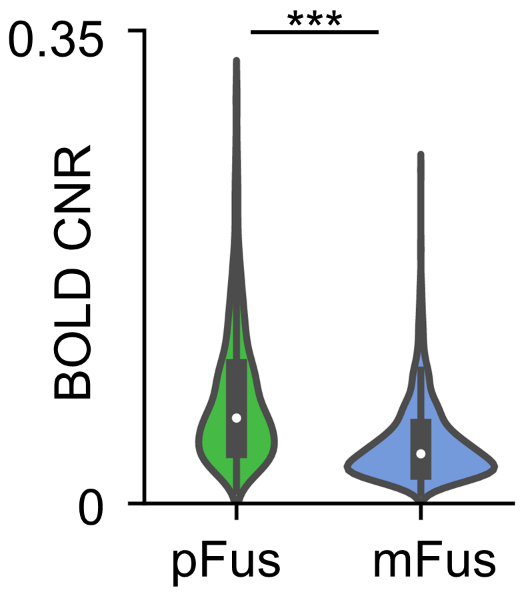


**Fig. S1. pFus-faces/FFA-1 and mFus-faces/FFA-2 showed significantly different BOLD CNRs.** ***p<0.001.


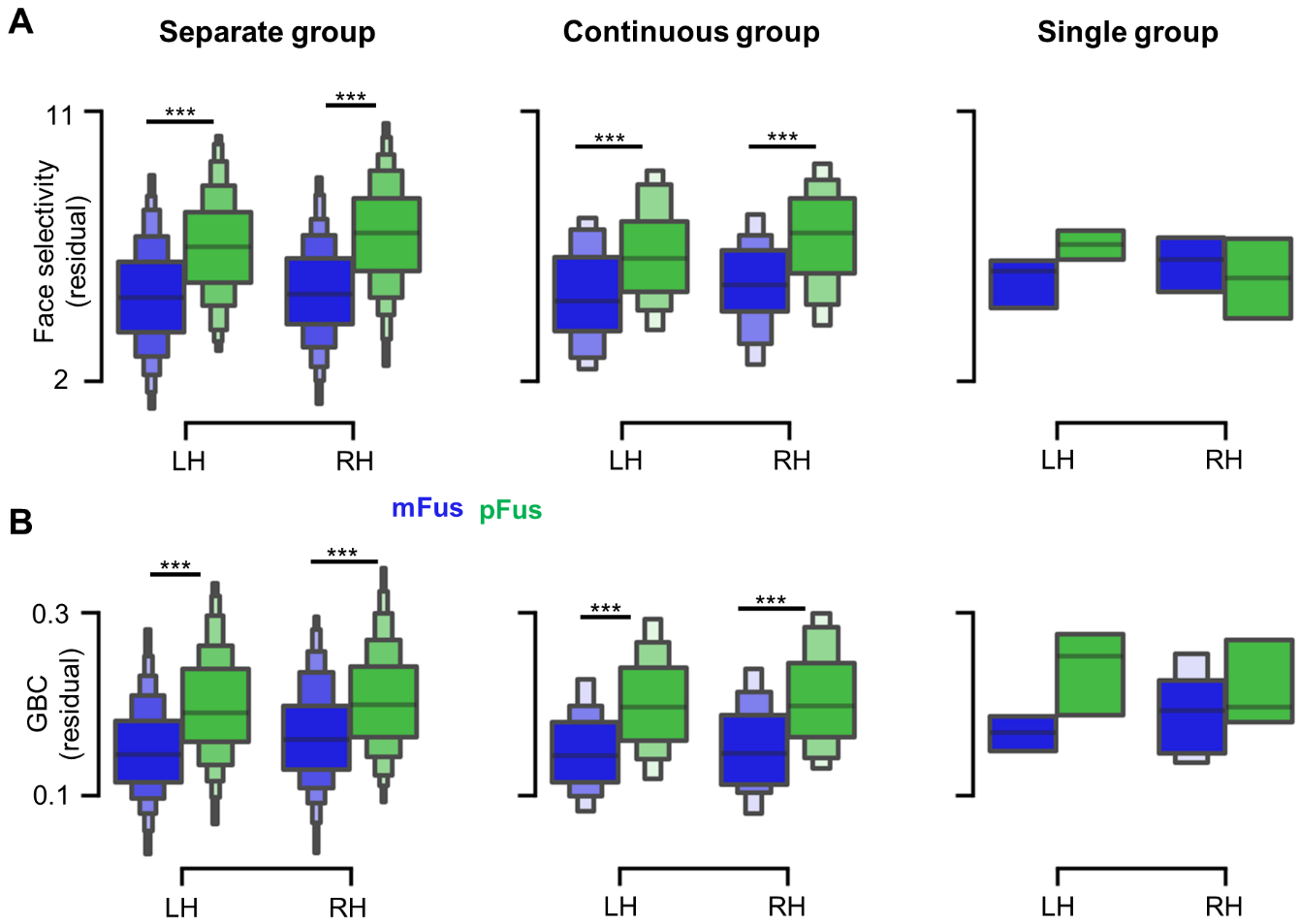


**Fig. S2. pFus-faces/FFA-1 and mFus-faces/FFA-2 still showed significant differences in both face selectivity (A) and functional connectivity (B) after accounting for the BOLD CNRs.** GBC: global brain connectivity; LH: left hemisphere; RH: right hemisphere. ***p<0.001.

*Average and individual maps of myelin and thickness*


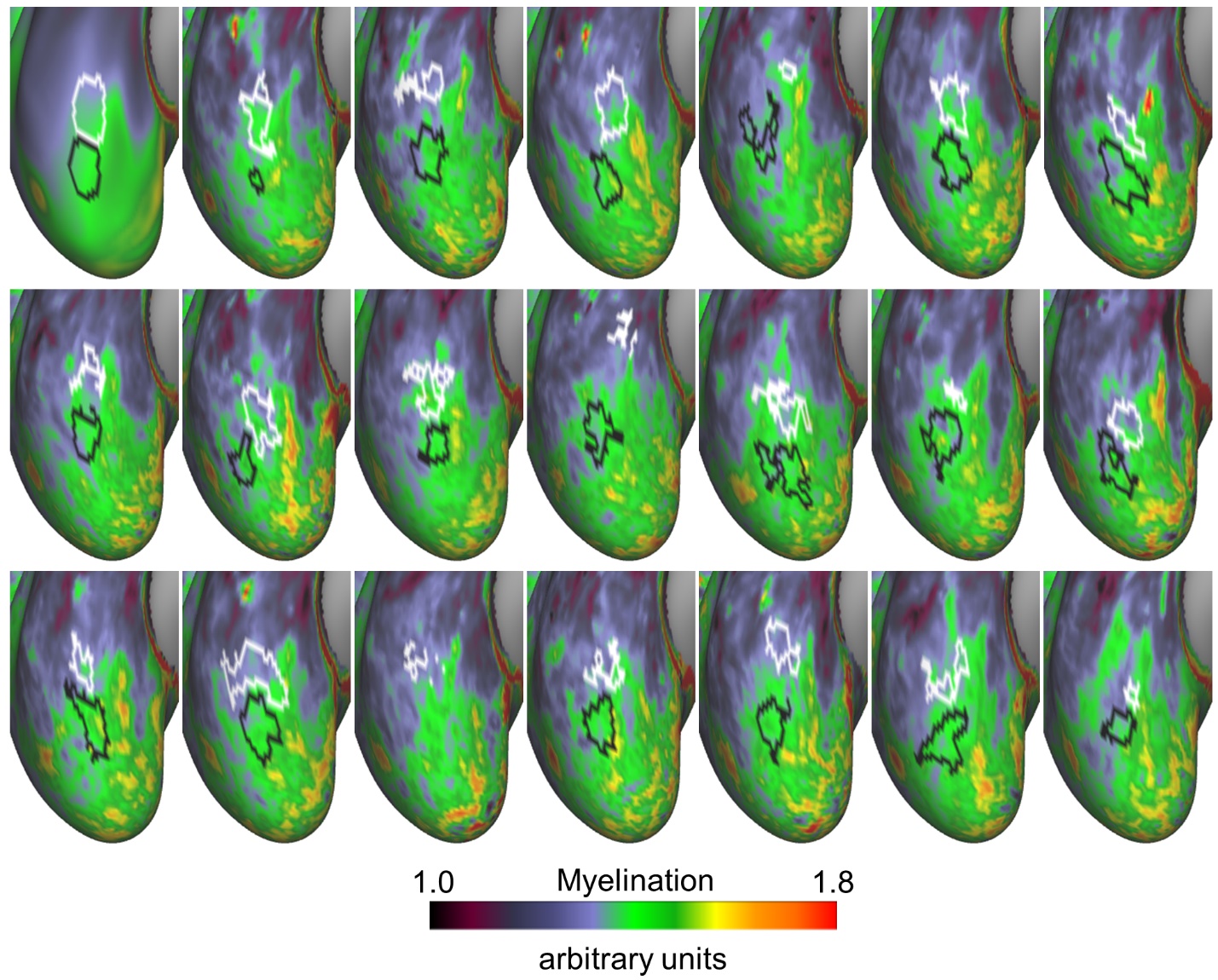


**Fig. S3. Group (upper left panel; averaged across 1053 hemispheres) and individual (other panels) myelin maps.** Lines overlaid on the maps are contours of group (maximum probability map) and individual pFus-faces/FFA-1 (black) and mFus-faces/FFA-2 (white).


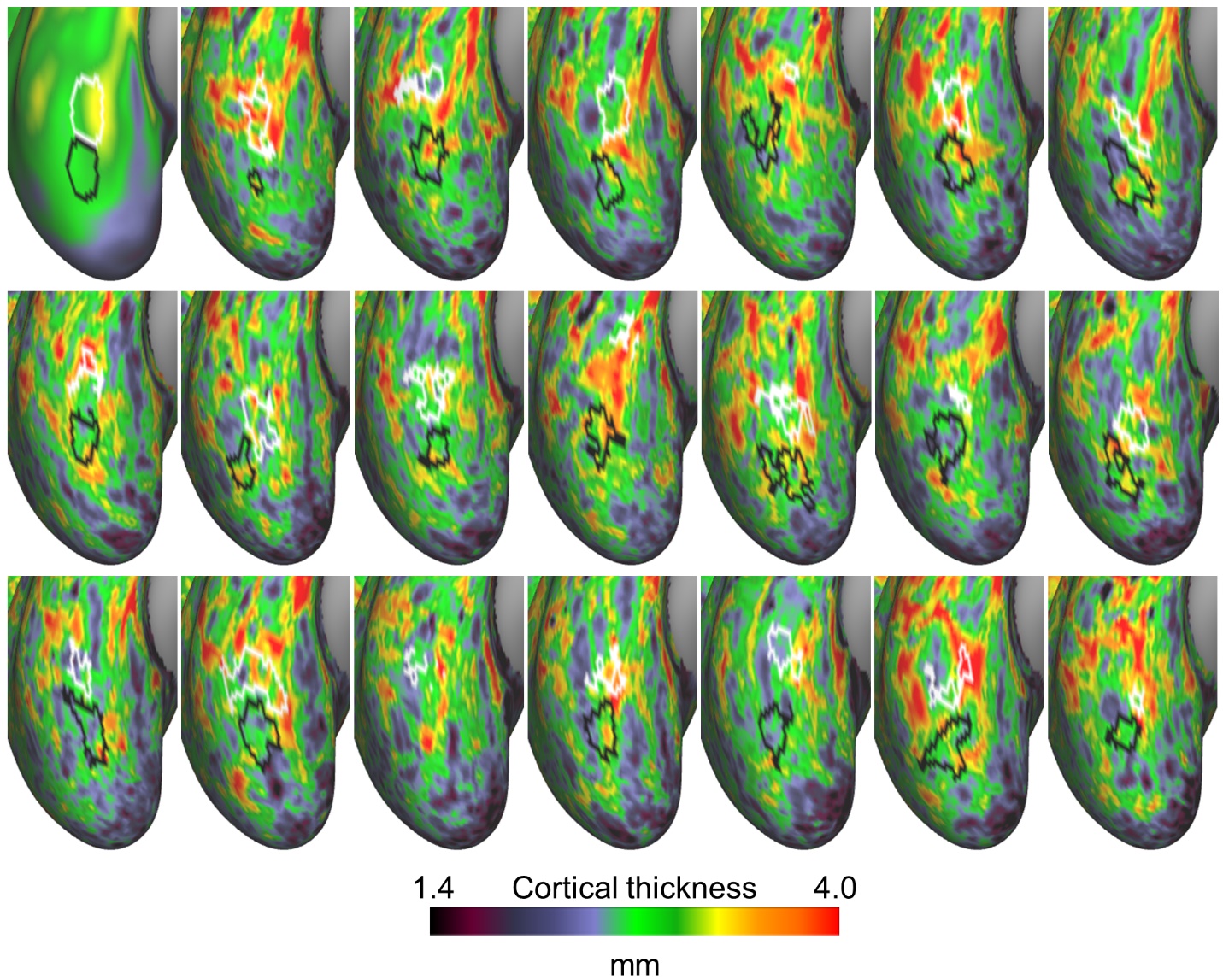


**Fig. S4. Group (upper left panel; averaged across 1053 hemispheres) and individual (other panels) cortical thickness maps.** Lines overlaid on the maps are contours of group (maximum probability map) and individual pFus-faces/FFA-1 (black) and mFus-faces/FFA-2 (white).

*Visualization of RSFC differences at the area level*

The Cohen’s D of the resting-state functional connectivity (RSFC) differences between pFus-faces/FFA-1 and mFus-faces/FFA-2 to 358 HCP multimodal parcellation (MMP) areas (Glasser et al., 2016; Except the left and right FFCs) was calculated and visualized on the inflated surface for the continuous group (Fig. S5) and the separate group (Fig. S6).


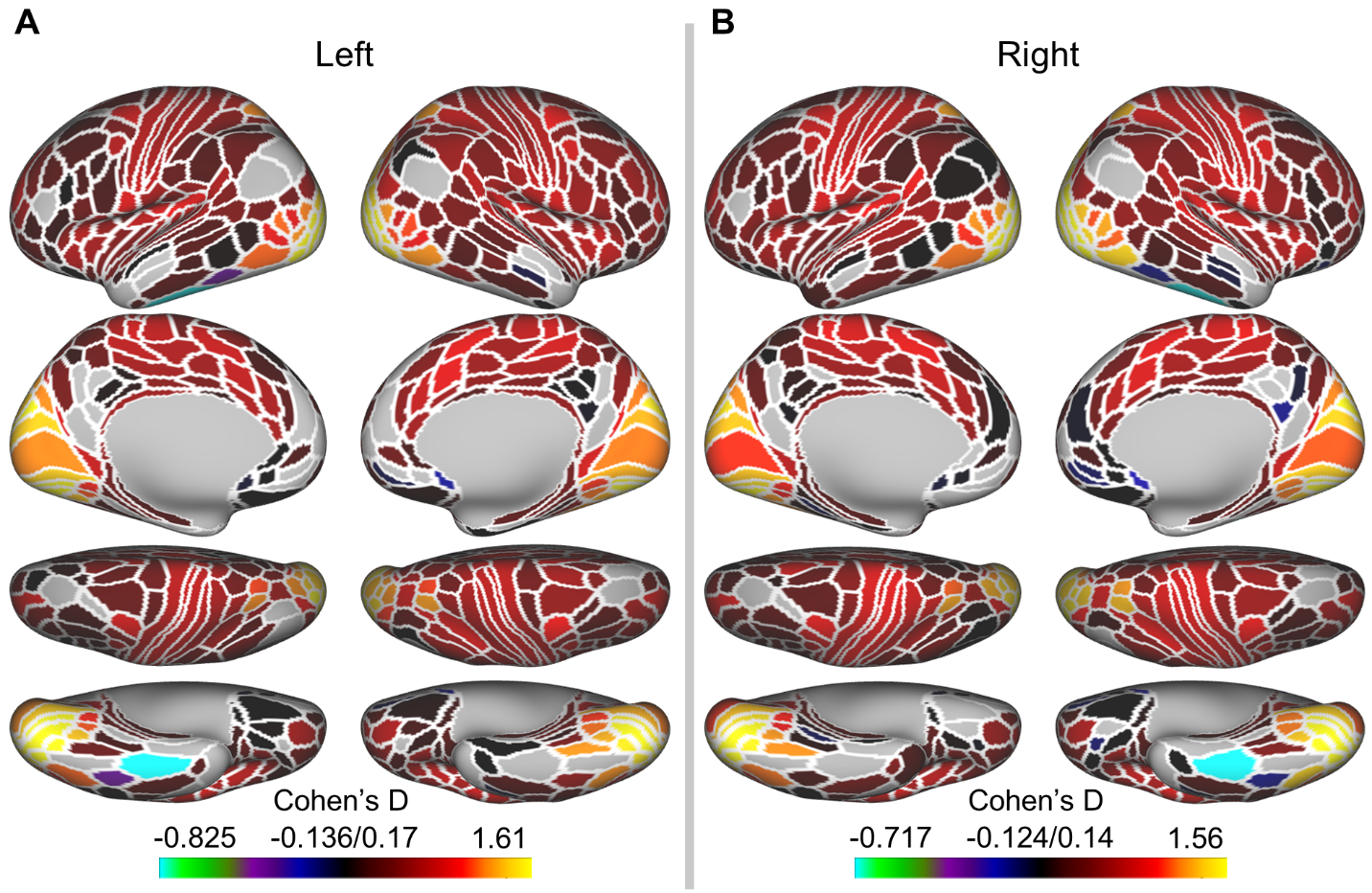


**Fig. S5. The RSFC differences between pFus-faces/FFA-1 and mFus-faces/FFA-2 to 358 MMP areas of the human cerebral cortex in the continuous group.** White lines indicate the boundary of HCP MMP areas. The areas that show significantly different RSFC to pFus-faces/FFA-1 and mFus-faces/FFA-2 (p<0.05, FDR corrected) are colored according to the Cohen’s D of the RSFC differences between the two FFAs, while others are displayed in gray (indicating non-significance). (A) RSFC is greater in left pFus-faces/FFA-1 compared to left mFus-faces/FFA-2 for a majority of areas. Left mFus-faces/FFA-2 shows stronger RSFC than left pFus-faces/FFA-1 in 8 areas: R_v23ab, R_10v, R_TE1a, R_25, L_TF, L_TE2p, L_25, and L_s32. (B) RSFC is greater in right pFus-faces/FFA-1 compared to right mFus-faces/FFA-2 for a majority of areas. Right mFus-faces/FFA-2 shows stronger RSFC than right pFus-faces/FFA-1 in 18 areas: R_7m, R_v23ab, R_10r, R_47m, R_9m, R_10v, R_TE1a, R_TF, R_TE2p, R_25, R_s32, R_STSva, L_v23ab, L_47m, L_PGs, L_PHA2, L_25, and L_s32.


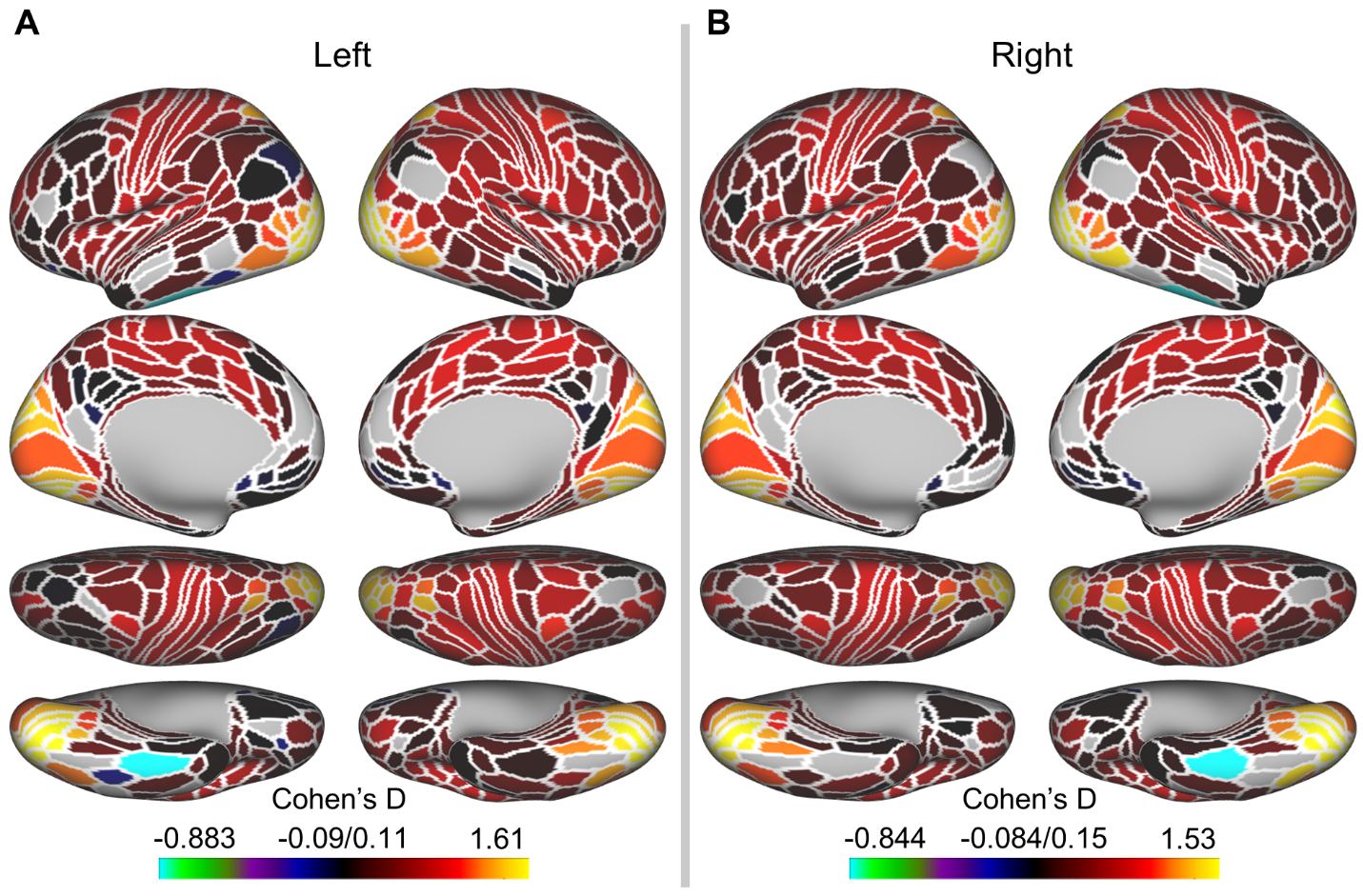


**Fig. S6. The RSFC differences between pFus-faces/FFA-1 and mFus-faces/FFA-2 to 358 MMP areas of the human cerebral cortex in the separate group.** Same layout as Supplementary Figure 5. (A) RSFC is greater in left pFus-faces/FFA-1 compared to left mFus-faces/FFA-2 for a majority of areas. Left mFus-faces/FFA-2 shows stronger RSFC than left pFus-faces/FFA-1 in 17 areas: R_v23ab, R_10r, R_10v, R_TE1a, R_25, R_s32, L_7m, L_v23ab, L_10r, L_47m, L_8Ad, L_10v, L_TF, L_TE2p, L_PGs, L_25, and L_s32. (B) RSFC is greater in right pFus-faces/FFA-1 compared to right mFus-faces/FFA-2 for a majority of areas. Right mFus-faces/FFA-2 shows stronger RSFC than right pFus-faces/FFA-1 in 7 areas: R_v23ab, R_10r, R_10v, R_TF, R_25, R_s32, and L_25.

*Supplementary References*

Glasser, M. F., Coalson, T. S., Robinson, E. C., Hacker, C. D., Harwell, J., Yacoub, E., Ugurbil, K., Andersson, J., Beckmann, C. F., Jenkinson, M., Smith, S. M., & Van Essen, D. C. (2016). A multi-modal parcellation of human cerebral cortex. *Nature*, *536*(7615), 171–178. https://doi.org/10.1038/nature18933

Weiner, K. S., & Grill-Spector, K. (2012). The improbable simplicity of the fusiform face area. *Trends Cogn. Sci.*, *16*(5), 251–254. https://doi.org/10.1016/j.tics.2012.03.003

Weiner, K. S., & Grill-Spector, K. (2013). Neural representations of faces and limbs neighbor in human high-level visual cortex: Evidence for a new organization principle. *Psychol. Res.*, 77(1), 74–97. https://doi.org/10.1007/s00426-011-0392-x
